# Effect on hand kinematics when using assistive devices during activities of daily living

**DOI:** 10.7717/peerj.7806

**Published:** 2019-10-08

**Authors:** Alba Roda-Sales, Margarita Vergara, Joaquín L. Sancho-Bru, Verónica Gracia-Ibáñez, Néstor J. Jarque-Bou

**Affiliations:** Departamento de Ingeniería Mecánica y Construcción, Universitat Jaume I, Castelló de la Plana, Spain

**Keywords:** Assistive device, Activities of daily living, Hand kinematics, Hand posture, Instrumented glove

## Abstract

Assistive devices (ADs) are products intended to overcome the difficulties produced by the reduction in mobility and grip strength entailed by ageing and different pathologies. Nevertheless, there is little information about the effect that the use of these devices produces on hand kinematics. Thus, the aim of this work is to quantify this effect through the comparison of kinematic parameters (mean posture, ROM, median velocity and peak velocity) while performing activities of daily living (ADL) using normal products and ADs. Twelve healthy right-handed subjects performed 11 ADL with normal products and with 17 ADs wearing an instrumented glove on their right hand, 16 joint angles being recorded. ADs significantly affected hand kinematics, although the joints affected differed according to the AD. Furthermore, some pattern effects were identified depending on the characteristics of the handle of the ADs, namely, handle thickening, addition of a handle to products that initially did not have one, extension of existing handles or addition of handles to apply higher torques. An overview of the effects of these design characteristics on hand kinematics is presented as a basis for the selection of the most suitable AD depending on the patient’s impairments.

## Introduction

Ageing and different pathologies reduce hand mobility and grip strength, affecting the normal performance of activities of daily living (ADL) (Brand & Hollister, 1999) and thus limiting personal independence. To overcome these difficulties, there are different commercially available assistive devices (ADs). According to the WHO (World Health Organization, 2019) ADs are those devices and technologies whose primary purpose is to maintain or improve an individual’s functioning and independence to facilitate participation and to enhance overall well-being. Bauer, Elsaesser & Arthanat (2011) propose a classification of ADs in health state ADs (HS ADs) and health-related states (HRS ADs). While the HS ADs aim to increase the level of functioning within a health domain which pertains to an individual’s body functions and body structures (e.g., a corneal implant), the HRs ADs are focused on health domains that pertain to an individual’s activities and participation (e.g., screen magnifiers). Additionally, HRS ADs are sub-classified in disability-specific (e.g., screen readers) and cross-disability (e.g., accessible bathrooms). In particular, this work is focused on HRS cross-disability mobility ADs, intended to overcome difficulties to perform ADLs experienced by users with reduced mobility, lack of grip strength or sensorimotor impairments, among others. Since there are several types of HRS cross-disability mobility ADs (referring to them as simply ADs from now in the manuscript) aimed at helping to perform the same ADL, the prescription process is not an easy task, and therapists must make decisions based on their own clinical experience. Therefore, any information about the effect of the ADs is essential in order to ensure that the prescribed AD will help to overcome the user’s limitations and improve his/her quality of life. Nevertheless, there is still little quantifiable information about the effect produced by the use of ADs.

Several works in the literature have presented reviews of ADs from different fields such as feeding (Holt & Holt, 2011), personal care (Harman & Craigie, 2011; Hepherd, 2011) or mobility (Stowe, Hopes & Mulley, 2010), but they only offer qualitative descriptions of the products and the difficulties that are presumably overcome. The use of ADs during the performance of ADL has been studied mainly through user surveys or group discussions (Mann, Hurren & Tomita, 1993; Kraskowsky & Finlayson, 2001; Hoffmann & McKenna, 2004; Wielandt et al., 2006; Hemmingsson, Lidstrom & Nygard, 2009; Skymne et al., 2012), focused on the identification of the reasons for rejection. Several useful conclusions were drawn from these works, such as the importance of the occupational therapist’s involvement in selecting devices due to inadequate information about the patients (Mann, Hurren & Tomita, 1993) and also the importance of considering the user’s perceptions and opinions during the AD selection process in order to prevent non-use (Wielandt et al., 2006). Nevertheless, the results remain qualitative.

Little research has attempted to study the quantitative effect of using ADs on the hand and upper limb posture (Van & Steenbergen, 2007; Ma et al., 2008; Ma et al., 2009b; McDonald et al., 2016). These experimental studies were conducted on healthy subjects (McDonald et al., 2016) and on subjects with pathologies such as Parkinson (Ma et al., 2008; Ma et al., 2009a) or cerebral palsy (Van & Steenbergen, 2007). Some of these works studied the effect of thickening products’ handle (Van & Steenbergen, 2007; Ma et al., 2008; McDonald et al., 2016), while others were focused on varying products’ weight (Ma et al., 2009a). For patients with Parkinson’s disease (Ma et al., 2008) the ADs aimed to mitigate the effect of problems as joint rigidity or decreased hand aperture while in cerebral palsy patients the aim is to increase grasp stability, independent finger control and force control (Van & Steenbergen, 2007). In these studies, parameters such as hand posture (McDonald et al., 2016), arm posture (Van & Steenbergen, 2007; Ma et al., 2009a) or hand-arm posture (Ma et al., 2008) were analysed and some conclusions were drawn. The product handle diameter was found to affect the speed and smoothness of upper limb movement (Ma et al., 2008), evidencing a relationship between the product shape and hand kinematics. Furthermore, in patients with Parkinson’s disease the small or medium handle size increased the speed and smoothness (Ma et al., 2008) while in patients with cerebral palsy thicker handles increased velocity of performance (Van & Steenbergen, 2007). Moreover, the kinematics were also found to be affecting the perceived comfort, products that could be managed with higher speed and smoothness being better rated by the users (Ma et al., 2008). Nevertheless, the hand kinematic analysis performed in all these studies had important limitations. One of them only considered metacarpophalangeal and interphalangeal flexion angles, which were measured manually with an electrogoniometer while performing a static grasp representative of the AD usage (McDonald et al., 2016). The other works studied the smoothness or velocity of arm movement with a three-dimensional ultrasonic measuring system using a single marker attached to the wrist of the participant’s dominant hand, which only allowed the arm movement to be studied, excluding the hand (Van & Steenbergen, 2007; Ma et al., 2008; Ma et al., 2009a). In addition, the ADL considered in the above mentioned quantitative analyses were very limited, considering only the task of eating with a spoon (Van & Steenbergen, 2007; Ma et al., 2008; McDonald et al., 2016).

However, there is little information in the literature about the kinematics of all the hand joints during the entire task performance when using different ADs (owing to the wide variety of products available) in comparison with normal products. Kinematic parameters such as range of motion (ROM) or mean postures (Gates et al., 2015), as well as velocities and smoothness ratio (Igo Krebs et al., 1998; Bosecker et al., 2010) are essential to quantify the efficiency of the task performance and are therefore useful to assess the abilities of individuals with impairments or in rehabilitation processes.

A continuous record of all joints during the entire task performance is required for a representative study of the kinematics, which allows the calculation of velocities. Nevertheless, recording all hand joint angles simultaneously without affecting the normal use of products is challenging. In this respect, instrumented gloves or videogrammetry are the techniques most commonly used for hand posture analysis when the study of a large number of joints is intended. However, data acquisition with videogrammetry during ADL is not feasible because of the occultation and collision of markers, instrumented gloves being an alternative.

Therefore, the aim of this work is to analyze the effect of ADs on hand kinematics not only focusing on the ROM, but also on the mean postures and velocities. To do so, hand kinematics parameters (mean angle, ROM, median velocity and P95 velocity) of healthy subjects when using ADs during the performance of a variety of ADL (opening cans and bottle, pouring from a carton, drinking with a glass, eating with some spoons, using a fork, carrying a dish, using a tap, brushing teeth and sliding zips) are compared with the same parameters obtained when performing the same ADL with the standard product. This comparison might be useful to contribute to a better assessment of ADs depending on the pathologies or impairments that they are intended to supplement with their use, and it can also be helpful to understand ADs users’ reasons for rejection. In addition, the study of these effects may be useful during the process of designing new products intended to improve the daily living of patients with specific pathologies.

## Materials & Methods

Twelve healthy right-handed subjects (six male, six female; age 35 ± 9.17 years) volunteered to participate in the experiment, approved by the Universitat Jaume I ethical committee (UJI-27/05/15-DPI201452095P). The subjects were previously informed about the characteristics of the experiment and gave their written consent.

### Selection of tasks and material

**Figure 1:**
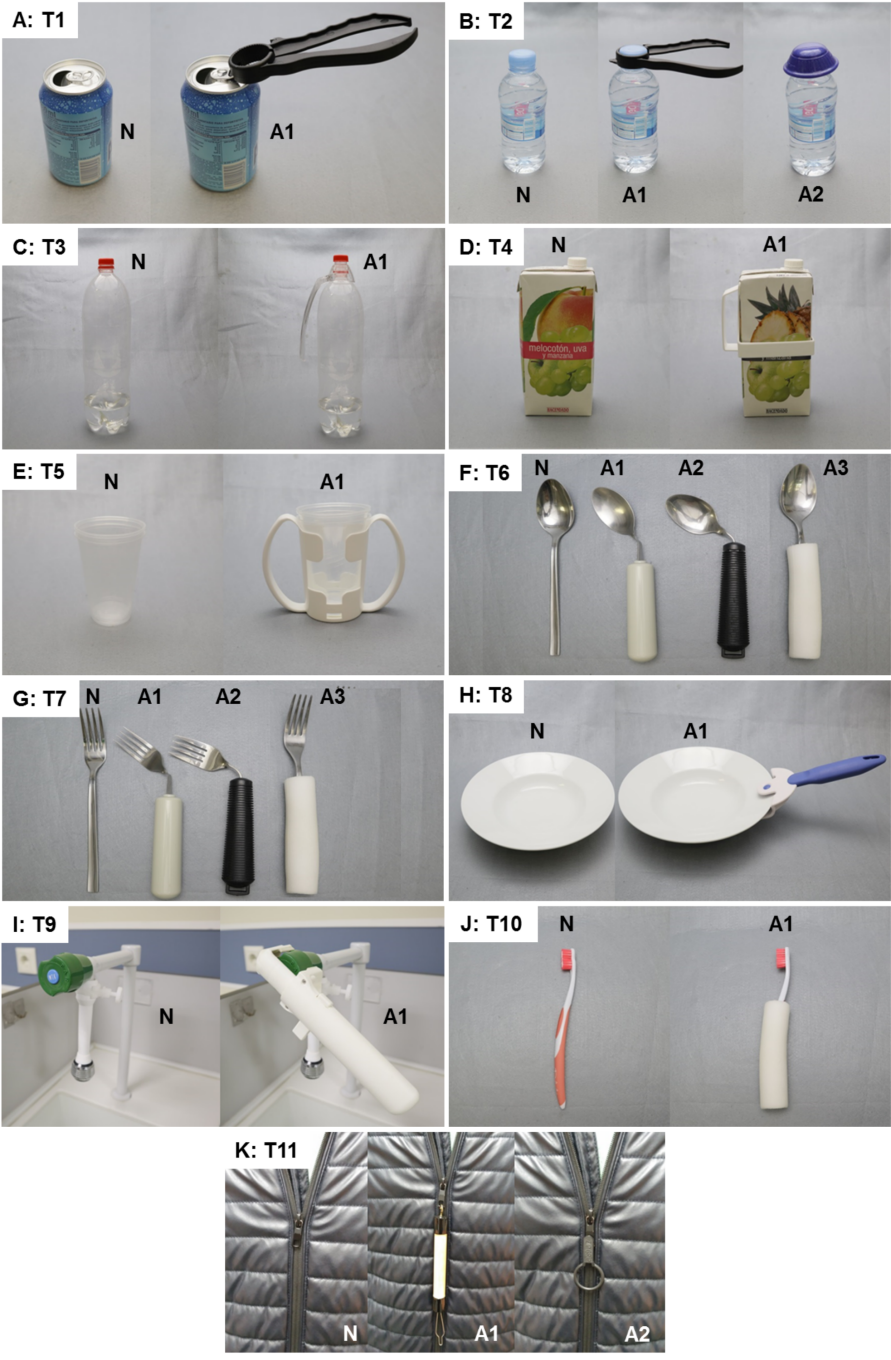
Products used during the performance of the ADL (tasks (A) T1 to (K) T11) considered in the experiment. The labels A1, A2 and A3 refer to the different assistive devices used for the task; the label N refers to the normal product.

The different typologies of commercially available ADs for grasping were studied (such as personal care, dressing, eating or drinking) and 17 products were chosen to be representative of those intended to solve hand mobility or strength limitations during product manipulation. Then, according to these ADs, 11 ADL associated with their use were selected. Table 1 presents the list of specific tasks and products selected and the body posture during each task performance. Tasks were carried out with the normal products and with one, two or three ADs (Fig. 1). Design characteristics of each assistive device are presented in Table 2.

**Table 1:** ADL performed in the experiment, products used and body posture during their performance.

ID	Task	Products	Posture
T1	Opening cans	1 NP, 1 AD	Sitting
T2	Unscrewing a bottle top	1 NP, 2 ADs	Sitting
T3	Pouring from a bottle	1 NP, 1 AD	Sitting
T4	Pouring from a carton	1 NP, 1 AD	Sitting
T5	Drinking from a glass	1 NP, 1 AD	Sitting
T6	Eating with a spoon	1 NP, 3 ADs	Sitting
T7	Eating with a fork	1 NP, 3 ADs	Sitting
T8	Carrying a dish	1 NP, 1 AD	Standing
T9	Using a tap	1 NP, 1 AD	Standing
T10	Brushing teeth	1 NP, 1 AD	Standing
T11	Sliding a zip up	1 NP, 2 ADs	Standing

**Notes.**

NP normal product AD assistive device

**Table 2:** Design characteristics of the assistive devices used in each task.

Task	Product	Characteristics
T1	A1	Handle to apply higher torque and reduce precision requirements when pulling the tin ring.
T2	A1	Additional handle to apply higher torque.
	A2	Rubber cap over the original cap, to improve grip.
T3	A1	Vertical additional handle to the bottle.
T4	A1	Vertical additional handle to the carton.
T5	A1	Vertical additional handles (both sides) to the glass.
T6, T7	A1	Thickened and bended plastic cylindrical handle (Φ = 30 mm, bent angle = 40°).
	A2	Thickened and bended rubber conical handle. (Φ_1_ = 33 mm, Φ_2_ = 24 mm bent angle = 60°)
	A3	Thickened sponge cylindrical handle (Φ = 30 mm).
T8	A1	Horizontal additional handle to the dish (section 31 mm × 24 mm).
T9	A1	Additional handle to apply higher torque (section 35 mm × 25 mm).
T10	A1	Thickened sponge cylindrical handle (Φ = 30 mm).
T11	A1	Cylindrical extension of the original zip (Φ = 16 mm).
	A2	Toroidal extension of the original zip.

Three scenarios were prepared (Scenario 1: A chair with the objects to perform the dressing tasks; Scenario 2: A table with the objects to perform the eating/drinking tasks; Scenario 3: A sink and a table with the objects to perform the self-care tasks). The objects were arranged in the same position for all the subjects, and the initial and final postures of each subject were controlled to ensure they were the same (hands and arms relaxed when standing, and with their hands lying relaxed on the table with the palm down when sitting).

### Experiment

**Figure 2:**
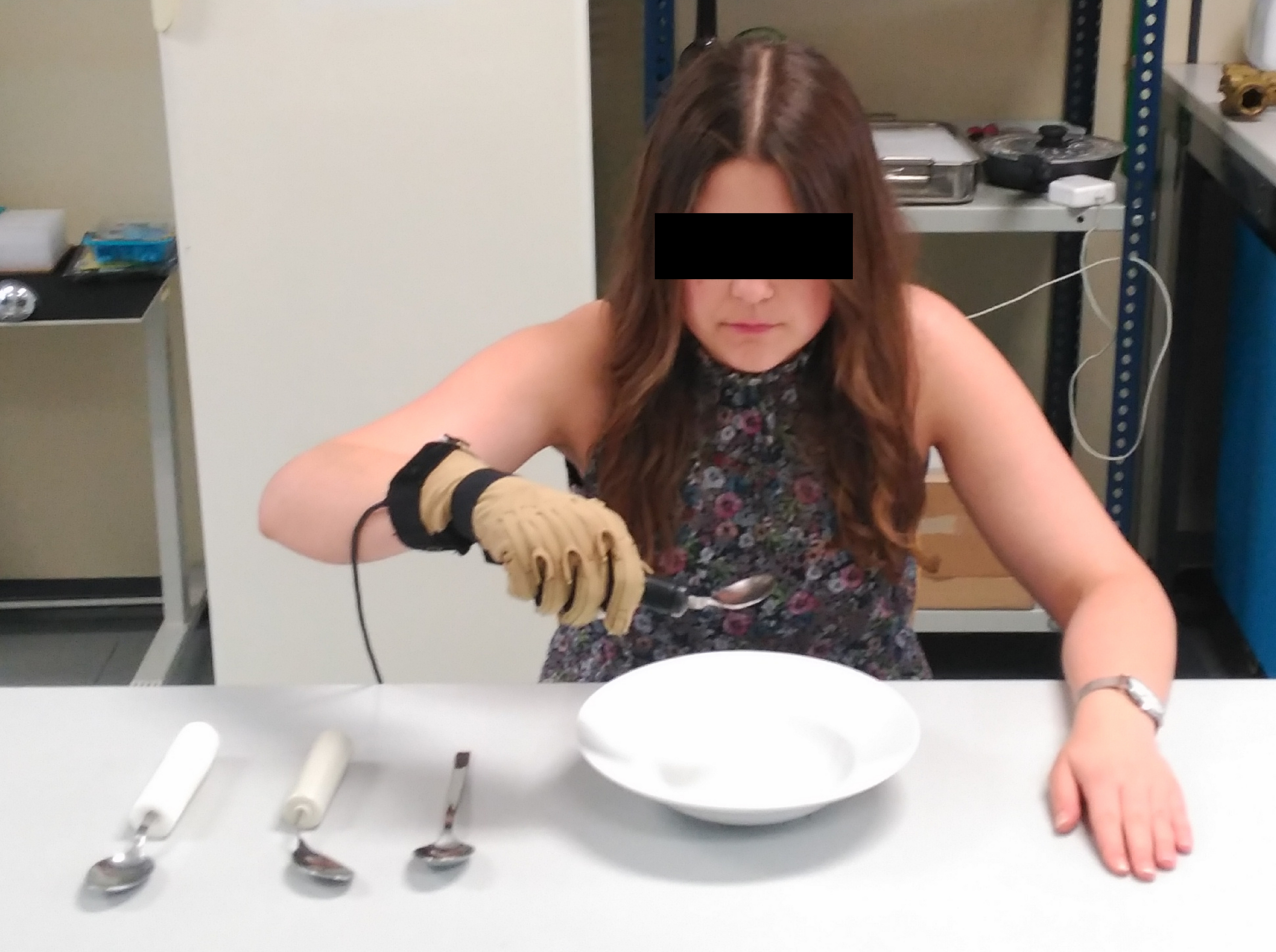
Subject wearing the instrumented glove performing the task of eating with a spoon.

**Figure 3:**
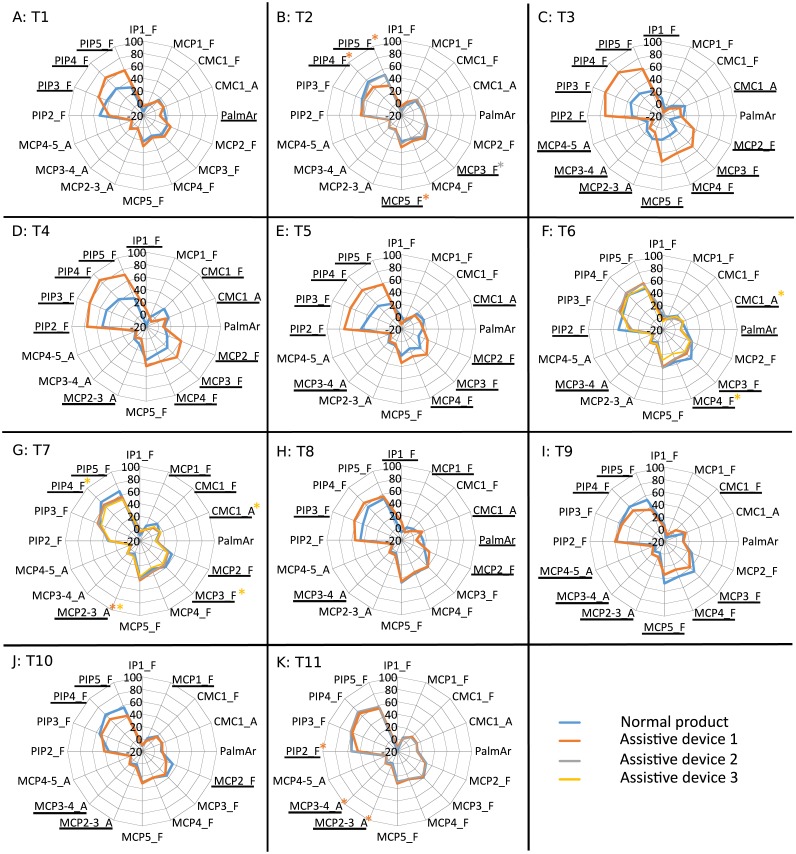
Mean values of the mean angle (deg) obtained for each joint, task ((A) T1 to (K) T11) and product. Joints with significant differences for all the ADs are underlined. Joints with significant differences for some ADs are underlined and marked with an asterisk of the corresponding colour. Tasks and products labelled as described in Table 1 and Fig. 1. Joints labelled as described in main text. Positive values for flexion, abduction of fingers and palmar deviation of thumb.

The subjects performed all the tasks wearing an instrumented glove CyberGlove^®^ (CyberGlove Systems, San José, CA, USA) on their right hand (Fig. 2), and 16 hand joint angles (described in caption of Fig. 3) were recorded. The tasks were performed with both hands when needed, although the products were always used with the right hand. The order of the tasks for each subject was randomized. In all, 336 (12 subjects ×  28 products) continuous records (acquired at a frequency of 100 Hz) of 16 gauges were the data collected while performing the tasks. Note that each record had a different duration.

### Data analysis

A previously validated protocol (explained in detail in Gracia-Ibáñez et al. (2017) was used to calculate 16 hand joint angles from the gauge data recorded by the CyberGlove. The protocol consists of computing gains and corrections to avoid cross-coupling effect by averaging those obtained from detailed subject-specific calibration involving 44 registrations. The angles were then low-pass filtered (2nd order Butterworth filter, cut-off frequency five Hz). After that, initial and final data of each record (while there was no movement detected) were discarded. The instant velocity of each joint was computed as the joint posture variation within the data sampling period (*T* = 0.01s, as the data acquisition frequency was 100 Hz). For each record and for each joint angle, four kinematic parameters were computed: mean angle, ROM (calculated from percentiles 5 to 95 of joint angles), median velocity and percentile 95 of velocity. The time taken to accomplish each task with each product was also computed. For each of the four kinematic parameters (mean posture, ROM, median velocity and P95 velocity) of each joint angle, 17 one-way repeated measures ANOVAs were conducted, considering as the factor for the ANOVA each of the 17 ADs against its corresponding normal product. In this way the effect of the type of product used (normal or AD) on kinematic parameters in each joint angle can be checked.

## Results

Regarding the analysis of postures, Fig. 3 presents the mean of the mean angles at each joint when performing the tasks with normal products and different ADs. Significant differences from the repeated-measures ANOVAs (bilateral asymptotic sig. ≤ 0.01) while performing tasks with normal products (N) and the different ADs available (A1, A2 and A3) are marked in each joint.

Joints studied were: thumb interphalangeal joint (IP1), carpometacarpal joint of thumb (CMC1), metacarpophalangeal joints (1 to 5, thumb to little digits) (MCP1 to MCP5), palmar arch (PalmAr), proximal interphalangeal joints (2 to 5, index to little digits) (PIP2 to PIP5), abduction between index and middle fingers (MCP2-3_A), abduction between middle and ring fingers (MCP3-4_A), and abduction between ring and little fingers (MCP4-5_A).

**Figure 4:**
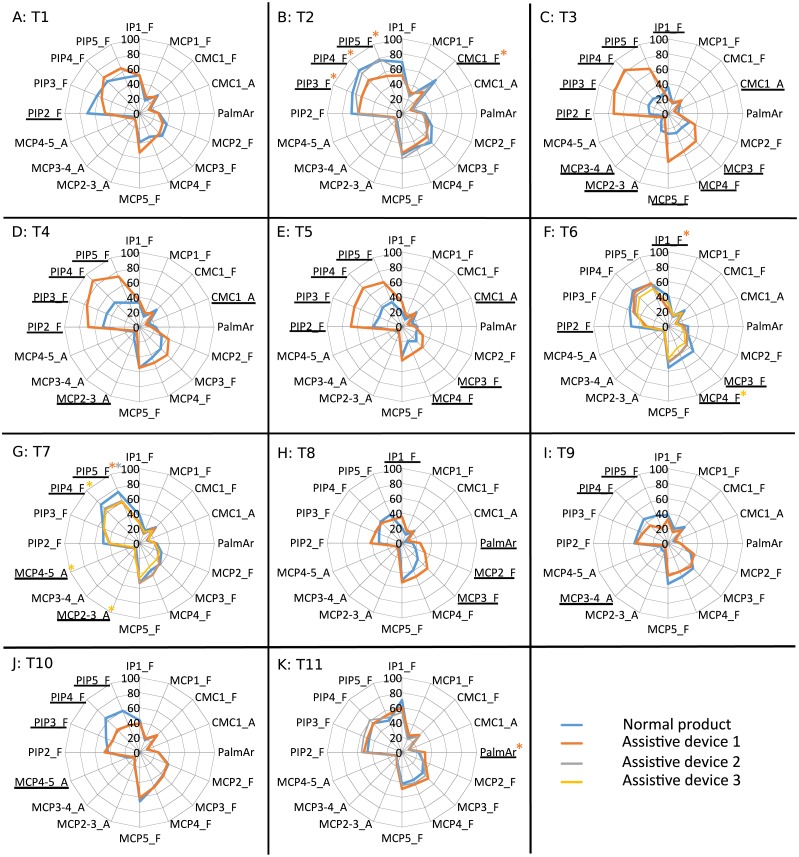
Mean values of the ROM (deg) obtained for each joint, task ((A) T1 to (K) T11) and product. Joints with significant differences for all the ADs are underlined. Joints with significant differences for some ADs are underlined and marked with an asterisk of the corresponding colour. Tasks, products and joints labelled as described in Fig. 3.

In addition, Fig. 4 presents the mean values of the ROM of each joint obtained when performing each task with normal products and each AD, where significant differences are also marked. Tables of mean values and standard deviation of mean postures and ROM when performed with normal products and ADs are presented as Supplemental Files.

Significant differences in posture and ROM are found in all the ADs analyzed, except for ROM of the bottle opener A2 (T2) and mean posture and ROM of the zip adapter A2 (T11). It can be observed that all the joint angles are affected by the use of some of the ADs.

**Figure 5:**
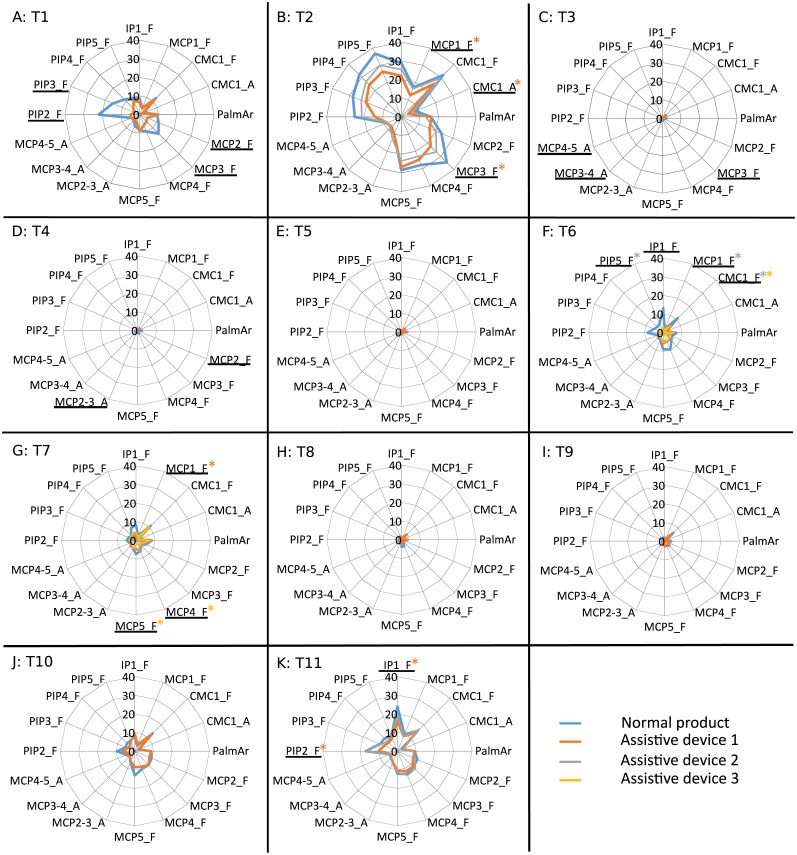
Mean of the median velocity (deg/s) obtained for each joint, task ((A) T1 to (K) T11) and product. Joints with significant differences for all the ADs are underlined. Joints with significant differences for some ADs are underlined and marked with an asterisk of the corresponding colour. Tasks, products and joints labelled as described in Fig. 3.

**Figure 6:**
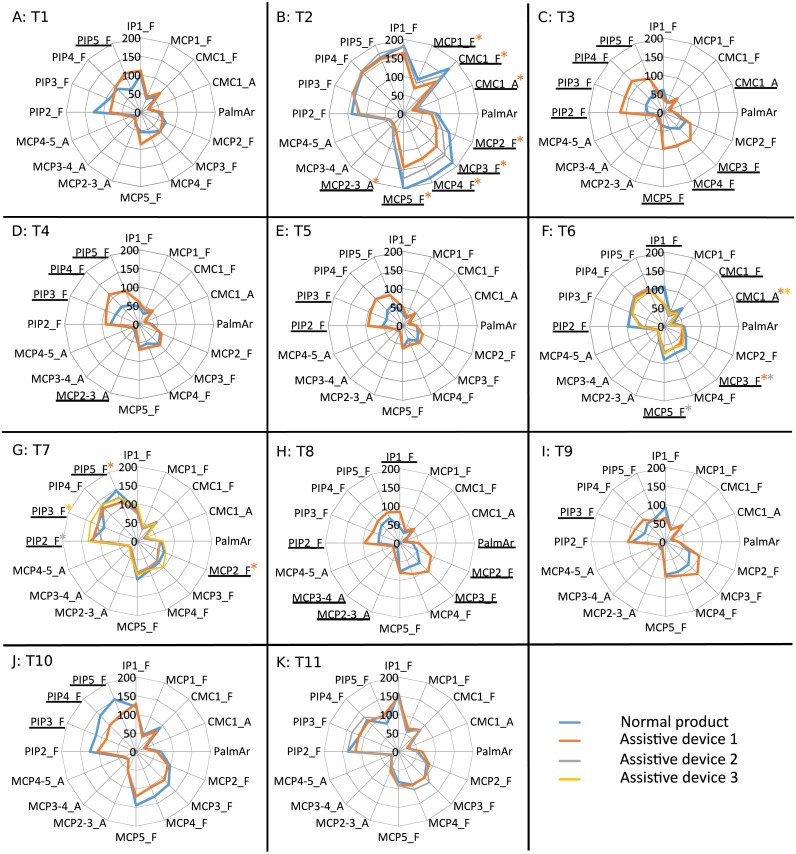
Mean values of the percentile P95 values of velocities (deg/s) obtained for each joint, task ((A) T1 to (K) T11) and product. Joints with significant differences for all the ADs are underlined. Joints with significant differences for some ADs are underlined and marked with an asterisk of the corresponding colour. Tasks, products and joints labelled as described in Fig. 3.

Regarding the analysis of velocities, Fig. 5 presents the mean values of the median velocities at each joint obtained when performing the tasks with normal products and ADs, and Fig. 6 presents the mean values of the P95 in the same way. Significant differences (bilateral asymptotic sig. ≤ 0.01) found in the repeated-measures ANOVAs are marked in both figures. Tables of mean values and standard deviation of median velocities and peak velocities when performed with normal products and ADs are presented as Supplemental Files.

In general, all the significant differences found in median velocity imply a reduction, and differences are obtained for almost all joints and movements.

**Figure 7:**
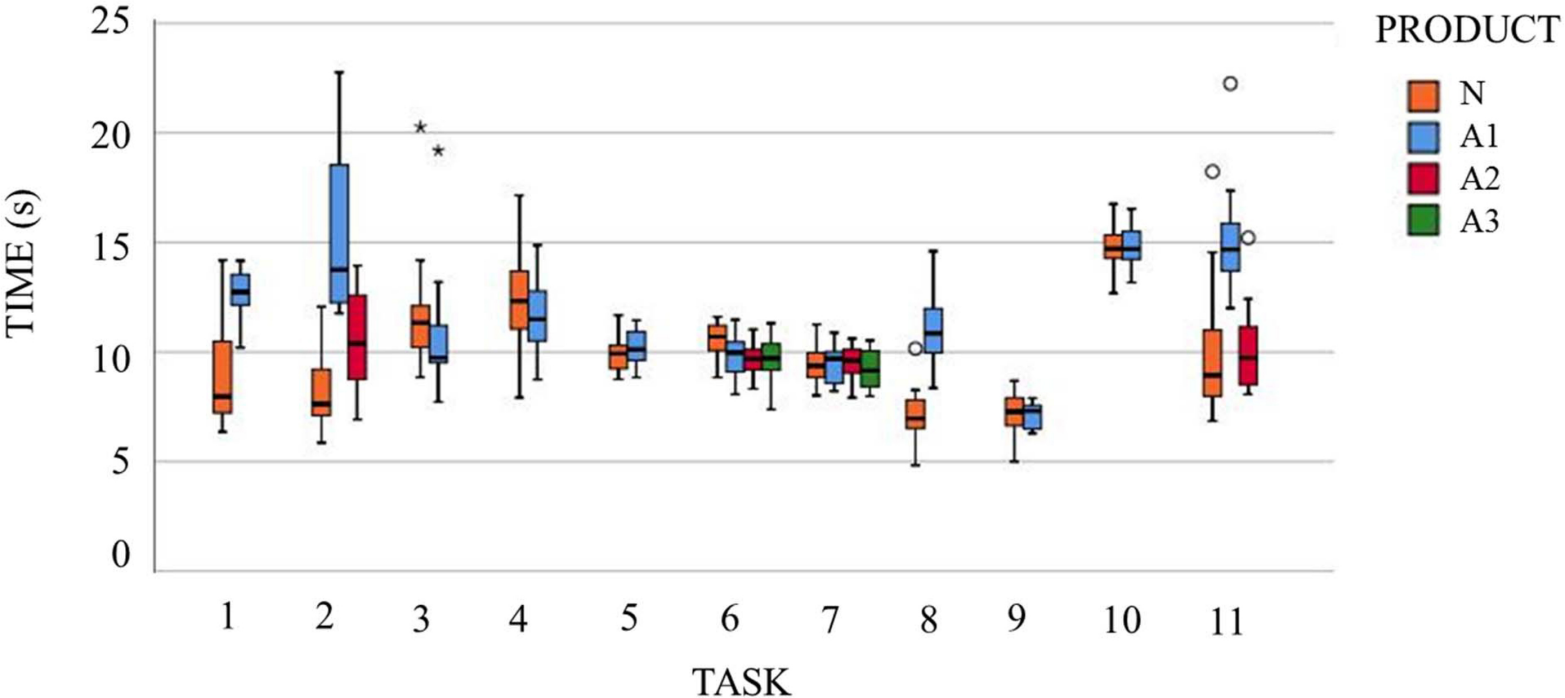
Box-plots of time of accomplishment of the tasks when performed with the different products. Tasks and products labelled as described in Table 1 and Fig. 1.

Figure 7 shows the box-and-whiskers plot of the time of accomplishment of each task when performed with the normal product and with the different ADs.

After applying a repeated-measures ANOVA to the time of accomplishment, significant differences (bilateral asymptotic sig. ≤ 0.01) are found for the tasks of opening a can (T1), unscrewing a bottle top (T2), pouring from a bottle (T3), eating with a spoon (only for A2 and A3) (T6), carrying a dish (T8) and sliding a zip up (T11) (only for the A1 adapter). It can be observed that the time of accomplishment in all of these tasks with differences is far higher when performed with ADs except for the task of pouring from a bottle and eating with a spoon.

## Discussion

Firstly, in order to assess the goodness or disadvantage of the effects observed in the posture analysis, it is important to analyze whether they favor using a more neutral or awkward (extreme) posture. In this sense, the significant increase in flexion at the thumb interphalangeal (IP) joint by some of the ADs considered leads to a more comfortable posture, as they allow the user to move away from a posture that is too extended. In fact, in some pathologies, such as stroke, the subject finds it difficult to perform digit extension, and this is considered as an indicator of recovery (Smania et al., 2007). The observed increase in flexion in proximal interphalangeal (PIP) and metacarpophalangeal (MCP) joints in tasks such as pouring from a bottle (T3), pouring from a carton (T4) and drinking from a glass (T5) is more critical than that observed in tasks like opening a can (T1), since the mean postures used with the ADs are far less neutral in the first ones. Looking at finger abduction, the tap adapter (T9) generates an increase in abductions of fingers index to little, while the bottle adapter (T3) gives rise to a reduction. An increase in abduction between middle and ring fingers when using the AD spoons (T6) and between index and middle fingers when using the adapted forks A1 and A3 (T7) is also observed. Nevertheless, these increases may not imply any negative effect since in all cases the joint angles when using ADs are lower than 10 deg. In particular, the use of the AD for the task of using a tap (T9) solves the negative abduction values required with a normal product, which implies a less neutral posture. The significant differences obtained for the thumb flexion show coordination between the MCP and carpometacarpal (CMC) joints. Looking at flexion of the palmar arch and the thumb CMC joint, significant differences always correspond to a decrease in these joint angles when using the ADs, except for the tap adapter (T9). This can be attributed to the general thickening of the handles in ADs and the shape modulation function of the palmar arch during grasping (Sangole & Levin, 2008), which is translated into more neutral postures for this joint complex.

The ROM results allow the posture analysis to be completed. Although significant differences are found for the ROM when using any of the ADs, fewer joint angles are affected in comparison to those affected when looking at mean postures. For the analysis, it is important to have in mind that high ROM values make manipulation difficult in patients with reduced hand mobility. Some of the ADs decrease the ROM of all the joints that are significantly affected: the can opener (T1), the bottle opener A1 (T2), all the AD spoons (T6), the AD forks A1 and A2 (T7), the tap adapter (T9) and the toothbrush adapter (T10). However, the differences obtained when using the dish adapter (T8) and the zip adapter A2 (T11) only imply increases in the ROM. And other ADs increase the ROM at some joint angles while they decrease it at others. However, these increases are not critical when looking at the final ROM values used with the ADs, except for pouring from a bottle (T3), pouring from a carton (T4) and drinking from a glass (T5), which may be a problem for patients with pathologies presenting reduced mobility, such as osteoarthritis or rheumatoid arthritis.

A detailed analysis by task allows us to identify some groups of tasks providing similar posture outcomes. It can be clearly seen that there is an increase in flexion of all the PIP joints when using the bottle adapter (T3), the carton adapter (T4) and the glass adapter (T5), with higher flexion ROM at the PIP joints, and lower abduction ROM at the thumb CMC joint. These results are coherent since all these adapters consist in adding a handle to the objects to be grasped, thus reducing the effective grasping diameter (a handle is grasped instead the object itself). All the spoons (T6) present lower flexion of the palmar arch, middle MCP and index PIP joints, and higher abduction between middle and ring fingers, along with ROM reduction of flexion at the middle MCP joint and index PIP joint. Furthermore, all the forks (T7) reduce the flexion of the thumb MCP, thumb CMC, index MCP and little PIP joints and forks A1 and A3 increase the abduction between index and middle fingers, thereby reducing the ROM of flexion of little PIP joint. The results obtained for the spoons are coherent with previous works focused on the effect of spoon handle diameter on ROM, finding that performing feeding tasks with spoons with thicker handles required lower ROM (McDonald et al., 2016). Owing to the similarity of the handles of the AD spoons and that of the AD toothbrush (and also the similarity of the grasp performed in both tasks), it can be extrapolated that for this product a lower ROM would also be required, which is also coherent with the results obtained.

A detailed analysis of velocities by task reveals two groups of products with clear patterns of changes that can be associated to their shape and design. The first group, composed of products with designs that add additional handles to products that initially did not have one (or significantly extend the existing ones), shows a general increase in peak velocities: the dish adapter (T8) (additional horizontal handle) or the tap adapter (T9) (additional handle to apply higher torque). Another finding worth noting is the increase in the peak velocity produced by those products with additional vertical handles (bottle adapter (T3), carton adapter (T4) and glass adapter (T5)) on almost all the PIP joints. Conversely, for the products with designs that involve thickened handles (AD spoons (T6) and toothbrush (T10)), or just a wider hand opening (bottle opener A1 (T2)), a general decrease in peak velocities is observed. It is notable that the decrease in median velocities found when using AD spoons (affecting the flexion rate in thumb IP and CMC) may be produced by the significant decreases obtained in peak velocities of the flexions in almost the same joints (index PIP, thumb IP and CMC). Therefore, a relationship can be established between the product diameter and the peak velocities, owing to the fact that the product diameter to be grasped is generally reduced in the ADs where an additional handle is added (carton, bottle, glass, dish, tap), with the exception of the zip adapter. This relationship is supported by the results obtained from ROM and P95 velocities analysis, and the relationship previously found between ROM and handle diameter. While in products such as the bottle opener A1 (T2), spoons (T6) and toothbrush (T10) a general decrease in ROM and P95 is found, in others such as the dish (T8) (and also in all the PIPs for the bottle (T3), carton (T4) and glass adapters (T5)), a general increase in both parameters is found. Nevertheless, these last tasks were initially found to be performed with almost static grasps, which means that these increases in velocities may take place during the phase of reaching for the product, rather than during manipulation.

It can be observed from Fig. 5 that when using normal products the hand is almost static in some tasks because the movement is being performed by the shoulder, elbow and/or wrist, as in the cases of pouring from a bottle (T3), pouring from a carton (T4), drinking from a glass (T5), carrying a dish (T8) and using a tap (T9). But other tasks present higher median velocity values, especially the task of unscrewing a bottle top (T2), which presents the highest values for all the joints. These median velocities are coherent with the type of grasp and manipulation performed during these tasks (in the aforementioned tasks, static grasps are usually performed, while in the task of opening a bottle a special grasp is performed combined with fine manipulation).

The analysis of velocities requires taking into account the peak velocities (Fig. 6). When using normal products, the highest values of peak velocities correspond to the unscrewing a bottle tap task (T2), and the ADs allow a significant reduction in these peak velocities. In general, the peak velocities of thumb decrease for the tasks of opening a bottle (with A1 opener) (T2) and eating with a spoon (T6), and increase in carrying a dish (T8). Regarding the MCP flexion, the values also decrease in the tasks of opening a bottle with A1 (T2) and eating with a spoon (T6), and increase when pouring from a bottle (T3) and carrying a dish (T8). The MCP abductions present fewer significant differences, the only remarkable ones being the reduction in the abduction between fingers when using the bottle opener A1 (T2) and the carton adapter (T4) or the increase when using the dish adapter (T8), only the differences found between index, middle and ring fingers being significant. Finally, a general increase in the PIP peak velocities is found in the tasks of pouring from a bottle (T3), pouring from a carton (T4) and drinking from a glass (T5).

As for the time of accomplishment analysis, the increase shown when using ADs can be attributable to the lack of experience of healthy subjects with this type of products. This increase in time of accomplishment is coherent with the general decrease in median velocities observed. Nevertheless, it is remarkable that for the task of eating with the spoons A2 and A3 (T6), apart from being performed in less time with ADs, all the significant differences in median and peak velocities imply a decrease in the values, which can be an indicator of grasp stability. In contrast, in the case of the dish adapter (T8), where time is higher and peak velocities increase significantly in the majority of joints, the smoothness of the movement is clearly lower. In this sense, a smoothness indicator can be determined from the difference between the peak and the median velocities, the lowest differences being taken as more beneficial. Thus, all those tasks with significant decreases in mean velocities and increases in peak velocities—the bottle adapter (T3) and the carton adapter (T4)—will have less movement smoothness. Additionally, low differences between peak and median velocities are observed in tasks such as opening a can (T2), brushing teeth (T10) and using a zip (T11), revealing a smooth operation. Nevertheless, these tasks are already smooth when performed with normal products, so it seems that no AD produces a significant improvement in movement smoothness.

**Figure 8:**
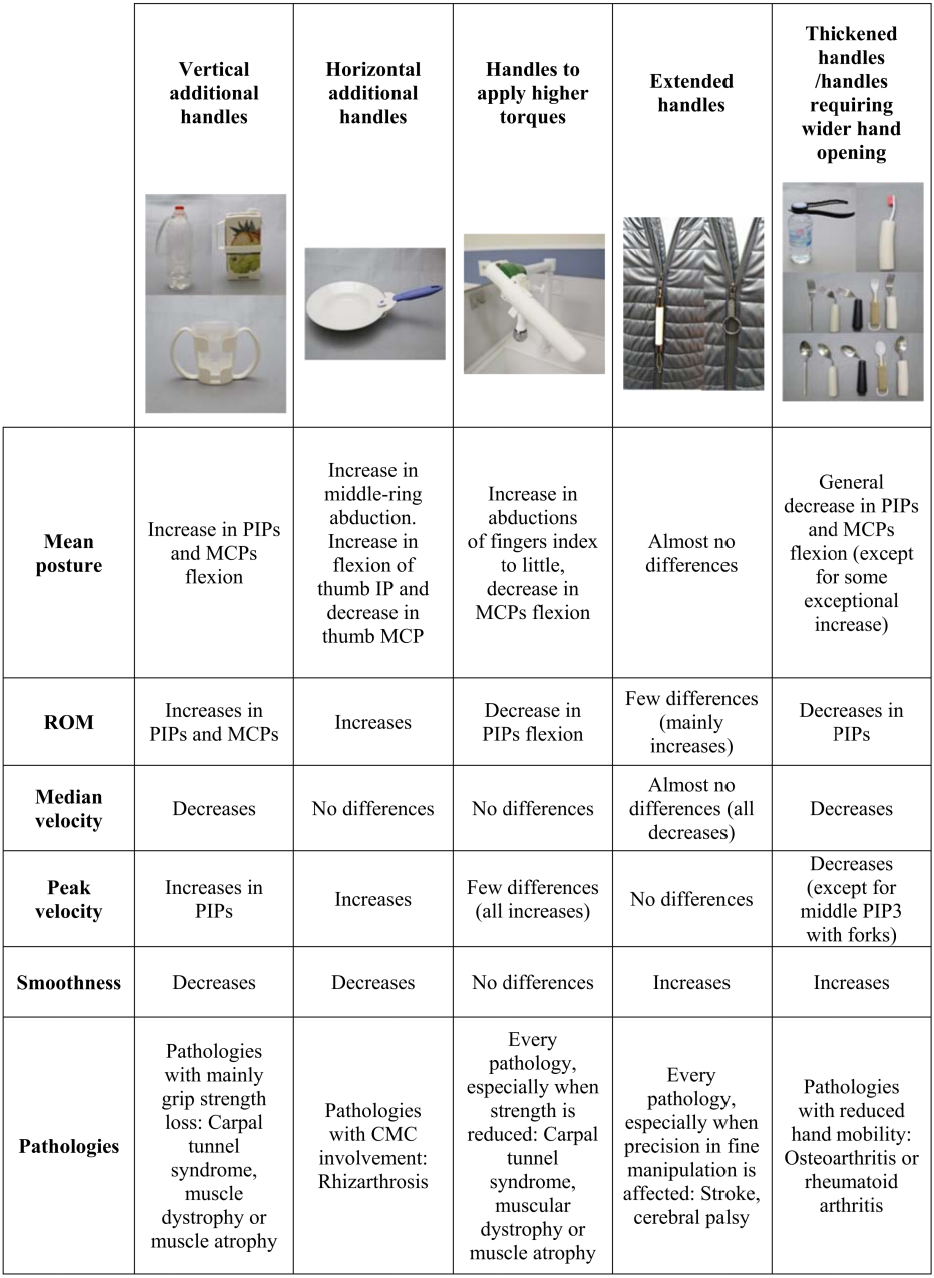
AD design characteristics and global results obtained across the different studies.

Overall, taking into account all the results of the different parameters and the design of the ADs, a five-group classification of ADs can be established: ADs with additional vertical handles (T3, T4, T5), with additional horizontal handles (T10), with handles to perform higher torques (T9), with extended handles (T11) and with thickened handles (A1 of T2, T6, T7 and T10). Figure 8 presents an overview of the global results obtained for each group from the different analyses of posture and velocity, as well as a brief example of pathologies that can benefit from the use of these ADs.

It can be observed that the design characteristics that imply the most changes are those with additional (vertical or horizontal) or thickened handles. The ADs that involve more neutral postures and lower velocities than the normal ones are those that have thickened handles or require a wider hand opening for the grasp, such as the bottle opener A1 (T2), the AD spoons (T6), the AD forks (T7) and the AD toothbrush (T10). However, the ones that consist in adding handles to products without one (the bottle adapter (T3), the carton adapter (T4), the glass adapter (T5) and the dish adapter (T8)) produce less neutral postures, higher velocities and less smoothness than the normal ones. These results may lead us to identify the latter as more suitable when strength is reduced and a grasp with more flexed PIPs and MCPs is needed in order to ensure that the object is not going to slip from the hand, but not for those patients with reduced hand mobility. Nevertheless, those with thickened handles may be in general more beneficial for all the pathologies, especially when hand mobility is reduced, since, in some studies, upper limb ROM analysis was found to allow therapists to assess the abilities of their patients (Gates et al., 2015), while peak and mean velocity were also commonly used when evaluating post-stroke patients (Alt Murphy & Häger, 2015). Smoothness was also identified as an important marker of patients’ motor recovery (Igo Krebs et al., 1998; Bosecker et al., 2010), as well as a parameter directly related with users’ rating when assessing the use of the product during studies focused on upper limb joints (Ma et al., 2008). Thus, in these cases special attention is needed when prescribing ADs to patients with the entire upper limb affected, since some products that help to overcome hand impairments may not be beneficial to the arm joints. Nevertheless, this work is an approach to AD assessment and the design of products and their kinematic implication should be studied individually before prescribing. Different pathologies may present different impairments such as reduced strength. In these cases, products with additional and thinner handles, despite increasing ROM or leading to less neutral postures, may be making performance of the task easier.

Finally, it has to be highlighted that the instrumented glove may introduce some loss of dexterity during the task performance that could have slightly affected the time of accomplishment of the tasks and also hand kinematics. Nevertheless, this loss of dexterity affects both the standard and the adapted product equally and, therefore, the comparison during the use of both types of products is valid. In addition, the experiments have been carried out on healthy subjects and may not reveal some of the difficulties experimented by patients during the performance of the tasks, but it was thought to be representative of the required kinematics under the best conditions. Performing more studies on subjects with specific pathologies may be useful in order to explore the similarity of results between healthy subjects and different pathologies. Moreover, it may reveal more accurate, but less general, effects.

## Conclusions

A detailed objective study of how hand kinematic parameters of healthy hands are affected when using different ADs during the performance of given tasks has been provided. Knowing how healthy hands are affected may help determine the most suitable ADs for a patient depending on the limitations derived from the pathology or the difficulties reported on daily living. The appropriateness of each AD has been shown to depend on the joints affected by the pathology, as not all the products affect the same joints in the same way, and they can reduce the ROM or improve mean postures for some joints, but lead to higher ROM or less neutral postures in others. Furthermore, an overview of the effects from using the ADs depending on the handle design has been provided, which makes the selection task considerably easier, thereby allowing therapists to prescribe the ADs objectively in a faster way. Moreover, this information about handle design implications could also be used by the AD manufacturers in order to ensure a better use of their products by establishing recommendations and precautions for use.

##  Supplemental Information

10.7717/peerj.7806/supp-1 Supplemental Information 1 Joint angles while performing the tasks using normal products and assistive devices The continuous calibrated recordings during the performance of each task using the different products, with filtered signal. Joint angles are presented in degrees. Tasks, products and joints are labelled as described in the manuscript.

10.7717/peerj.7806/supp-2 Supplemental Information 2 Mean values for posture, ROM, median velocity and peak velocity for each joint while performing the tasks using normal products and assistive devices

## Additional Information and Declarations

### Data Availability

The following information was supplied regarding data availability: The raw measurements are available as a Supplemental File.

## References

[ref-1] Alt Murphy M, Häger CK (2015). Kinematic analysis of the upper extremity after stroke—how far have we reached and what have we grasped?. Physical Therapy Reviews.

[ref-2] Bauer SM, Elsaesser LJ, Arthanat S (2011). Assistive technology device classification based upon the World Health Organization’s, International Classification of Functioning, Disability and Health (ICF). Disability and Rehabilitation: Assistive Technology.

[ref-3] Bosecker C, Dipietro L, Volpe B, Igo Krebs H (2010). Kinematic robot-based evaluation scales and clinical counterparts to measure upper limb motor performance in patients with chronic stroke. Neurorehabilitation and Neural Repair.

[ref-4] Brand PW, Hollister AM (1999). Clinical mechanics of the hand.

[ref-5] Gates DH, Walters LS, Cowley J, Wilken JM, Resnik L (2015). Range of motion requirements for upper-limb activities of daily living. American Journal of Occupational Therapy.

[ref-6] Gracia-Ibáñez V, Vergara M, Buffi JH, Murray WM, Sancho-Bru JL (2017). Across-subject calibration of an instrumented glove to measure hand movement for clinical purposes. Computer Methods in Biomechanics and Biomedical Engineering.

[ref-7] Harman D, Craigie S (2011). Gerotechnology series: toileting aids. European Geriatric Medicine.

[ref-8] Hemmingsson H, Lidstrom H, Nygard L (2009). Use of assistive technology devices in mainstream schools: students’ perspective. American Journal of Occupational Therapy.

[ref-9] Hepherd R (2011). Aids for bathing and showering. European Geriatric Medicine.

[ref-10] Hoffmann T, McKenna K (2004). A survey of assistive equipment use by older people following hospital discharge. British Journal of Occupational Therapy.

[ref-11] Holt RC, Holt RJ (2011). Gerotechnology: kitchen aids. European Geriatric Medicine.

[ref-12] Igo Krebs H, Hogan N, Aisen ML, Volpe BT (1998). Robot-aided neurorehabilitation. IEEE Transactions on Rehabilitation Engineering.

[ref-13] Kraskowsky LH, Finlayson M (2001). Factors affecting older adults’ use of adaptive equipment: review of the literature. American Journal of Occupational Therapy.

[ref-14] Ma H-I, Hwang W-J, Chen-Sea M-J, Sheu C-F (2008). Handle size as a task constraint in spoon-use movement in patients with Parkinson’s disease. Clinical rehabilitation.

[ref-15] Ma H-I, Hwang W-J, Tsai P-L, Hsu Y-W (2009a). The effect of eating utensil weight on functional arm movement in people with Parkinson’s disease: a controlled clinical trial. Clinical Rehabilitation.

[ref-16] Ma H-I, Hwang W-J, Tsai P-L, Hsu Y-W (2009b). The effect of eating utensil weight on functional arm movement in people with Parkinson’s disease: a controlled clinical trial. Clinical Rehabilitation.

[ref-17] Mann WC, Hurren D, Tomita M (1993). Comparison of assistive device use and needs of home-based older persons with different impairments. American Journal of Occupational Therapy.

[ref-18] McDonald SS, Levine D, Richards J, Aguilar L (2016). Effectiveness of adaptive silverware on range of motion of the hand. PeerJ.

[ref-19] Sangole AP, Levin MF (2008). Palmar arch dynamics during reach-to-grasp tasks. Experimental Brain Research.

[ref-20] Skymne C, Dahlin-Ivanoff S, Claesson L, Eklund K (2012). Getting used to assistive devices: ambivalent experiences by frail elderly persons. Scandinavian Journal of Occupational Therapy.

[ref-21] Smania N, Paolucci S, Tinazzi M, Borghero A, Manganotti P, Fiaschi A, Moretto G, Bovi P, Gambarin M (2007). Active finger extension: a simple movement predicting recovery of arm function in patients with acute stroke. Stroke.

[ref-22] Stowe S, Hopes J, Mulley G (2010). Gerotechnology series: 2. Walking aids. European Geriatric Medicine.

[ref-23] Van D, Steenbergen B (2007). The use of ergonomic spoons by people with cerebral palsy: effects on food spilling and movement kinematics. Developmental Medicine & Child Neurology.

[ref-24] Wielandt T, Mckenna K, Tooth L, Strong J (2006). Factors that predict the post-discharge use of recommended assistive technology (AT). Disability and Rehabilitation: Assistive Technology.

[ref-25] World Health Organization (WHO) (2019). Assistive devices and technologies. https://www.who.int/disabilities/technology/en/.

