# Supplementary material for: Effect on hand kinematics when using assistive devices during activities of daily living
Source: PeerJ. 2019 Oct 8;7:e7806. doi: 10.7717/peerj.7806 (PMC6788441; doi:10.7717/peerj.7806)
Supplement: Supplemental Information 2 [file peerj-07-7806-s002.pdf]

	IP1_F	MCP1_F	CMC1_F	CMC1_A	PalmAr	MCP2_F	MCP3_F	MCP4_F	MCP5_F	MCP2-3_A	MCP3-4_A	MCP4-5_A	PIP2_F	PIP3_F	PIP4_F	PIP5_F
**T1**	-13.0 (18.1)	-3.0 (7.5)	15.7 (15.3)	15.2 (3.5)	15.5 (7.9)	21.9 (10.5)	22.9 (10.4)	15.2 (11.9)	22.0 (12.0)	3.4 (4.4)	8.3 (3.7)	-0.3 (4.4)	50.4 (11.3)	43.2 (9.4)	41.5 (11.7)	28.9 (13.7)
**T2**	-14.1 (13.4)	-2.7 (6.0)	12.5 (14.4)	13.1 (1.9)	16.4 (10.4)	25.1 (9.9)	33.4 (11.2)	20.5 (11.1)	23.3 (12.4)	5.6 (4.2)	8.3 (2.5)	-0.1 (3.8)	44.3 (8.5)	48.6 (4.8)	56.0 (9.3)	50.1 (11.8)
**T3**	8.2 (10.2)	-8.9 (6.1)	2.6 (13.5)	20.4 (2.9)	14.6 (9.1)	-5.4 (8.3)	12.6 (13.9)	13.8 (12.1)	18.5 (10.5)	20.3 (8.7)	14.0 (5.1)	5.1 (4.4)	28.0 (15.7)	34.4 (7.4)	30.6 (9.0)	22.9 (10.6)
**T4**	-17.5 (13.5)	-3.1 (10.3)	20.7 (15.1)	18.5 (2.4)	9.3 (8.0)	16.2 (8.1)	27.9 (11.2)	26.6 (10.2)	33.8 (8.4)	10.1 (8.6)	7.4 (2.9)	-1.4 (3.5)	50.7 (14.7)	49.3 (13.4)	42.5 (16.2)	29.5 (17.9)
**T5**	0.7 (9.1)	-7.2 (5.6)	15.3 (16.5)	18.8 (2.6)	14.1 (10.8)	9.6 (11.5)	23.1 (13.0)	13.7 (10.3)	22.5 (9.4)	7.8 (5.2)	9.6 (1.7)	0.5 (5.0)	45.1 (9.1)	35.4 (6.8)	32.7 (9.2)	23.9 (9.1)
**T6**	3.0 (10.5)	2.6 (7.5)	12.3 (14.5)	13.6 (2.7)	17.5 (11.3)	30.7 (9.7)	44.9 (11.7)	35.4 (9.7)	40.6 (11.2)	2.0 (4.3)	6.6 (3.2)	-3.9 (3.1)	51.4 (10.5)	48.9 (8.3)	59.0 (13.4)	52.3 (17.0)
**T7**	-11.2 (18.4)	6.4 (8.2)	19.2 (10.7)	14.3 (3.0)	5.4 (10.4)	35.5 (6.2)	38.7 (11.6)	29.0 (11.6)	40.6 (12.8)	2.5 (3.3)	8.3 (2.5)	-5.1 (4.1)	30.8 (16.6)	49.2 (7.9)	68.3 (8.0)	66.5 (7.6)
**T8**	-7.0 (8.4)	2.5 (7.1)	6.0 (11.1)	11.4 (1.8)	14.7 (10.2)	20.5 (8.5)	40.4 (9.8)	36.7 (9.7)	48.0 (11.0)	2.6 (5.6)	5.3 (3.2)	-5.7 (3.3)	46.1 (12.0)	47.2 (14.2)	55.4 (18.1)	52.6 (19.1)
**T9**	6.9 (9.3)	-12.9 (7.8)	-8.4 (13.2)	13.8 (2.8)	10.2 (11.4)	27.7 (11.1)	47.8 (11.4)	40.5 (9.1)	47.1 (8.9)	-0.9 (6.9)	1.8 (3.2)	-5.0 (4.2)	57.3 (7.3)	56.2 (8.0)	59.4 (9.0)	52.7 (8.8)
**T10**	-11.2 (16.4)	3.1 (5.9)	14.5 (11.5)	14.0 (1.7)	10.7 (8.1)	32.9 (5.7)	35.3 (8.2)	25.9 (9.1)	30.2 (11.4)	2.7 (4.6)	7.5 (2.2)	-0.5 (4.4)	33.9 (8.4)	54.2 (9.4)	63.4 (8.5)	56.4 (8.1)
**T11**	-18.0 (15.5)	3.7 (8.6)	11.8 (13.7)	13.5 (1.9)	12.5 (5.7)	30.0 (8.5)	39.6 (7.4)	28.6 (7.2)	28.8 (10.2)	1.0 (3.6)	5.7 (2.6)	-0.7 (3.2)	54.0 (9.6)	58.9 (12.6)	68.4 (13.6)	55.4 (16.9)

Table S1: Mean (SD) of the mean angle (deg) for each joint and task performed with normal products. Joints, tasks and products labelled as in Figure 3. Positive values for flexion, abduction of fingers and palmar deviation of thumb.

		IP1_F	MCP1_F	CMC1_F	CMC1_A	PalmAr	MCP2_F	MCP3_F	MCP4_F	MCP5_F	MCP2-3_A	MCP3-4_A	MCP4-5_A	PIP2_F	PIP3_F	PIP4_F	PIP5_F
**T1**	**A1**	-4.9 (28.0)	-0.7 (10.5)	13 (13.7)	15.3 (3.8)	▼6.7 (12.1)	27.6 (7.1)	27.8 (14.9)	18.4 (16.9)	29.5 (20.5)	2.4 (4.2)	10.6 (2.6)	-0.5 (7.1)	35.1 (19.1)	▲57.6 (11.0)	▲66.1 (13.4)	▲58.8 (15.0)
**T2**	**A1**	-6.3 (10.4)	3.0 (5.3)	14.8 (14.2)	14.1 (1.6)	16.2 (7.6)	23.5 (9.1)	31.8 (9.9)	25.1 (7.5)	▲31.5 (6.8)	4.5 (3.3)	8.5 (3.3)	-0.2 (3.1)	45.4 (21.5)	45.5 (6.4)	▼44.2 (7.7)	▼31.3 (12.0)
	**A2**	-16.5 (17.0)	-1.9 (8.1)	16.6 (13.3)	13.3 (2.3)	15.2 (8.9)	22.0 (10.3)	▼28.4 (12.7)	19.0 (12.1)	26.4 (13.0)	6.5 (4.1)	9.3 (2.7)	-1.3 (3.6)	40.6 (7.0)	45.7 (6.3)	53.1 (10.8)	49.8 (12.5)
**T3**	**A1**	▼-5.7 (12.8)	-14.1 (7.9)	-3.3 (16.0)	▼12.0 (2.3)	10.6 (8.6)	▲35.4 (10.5)	▲49.4 (11.4)	▲44.6 (8.3)	▲53.6 (6.7)	▼8.9 (5.6)	▼7.9 (3.4)	▼-4.9 (3.8)	▲71.1 (25.6)	▲78.6 (7.5)	▲78.7 (7.1)	▲62.0 (9.5)
**T4**	**A1**	▲7.5 (17.3)	-4.6 (11.5)	▼-8.4 (17.5)	▼14.6 (3.5)	7.2 (7.9)	▲40.6 (11.0)	▲49.6 (11.6)	▲40.3 (9.8)	43.4 (11.5)	▼1.0 (4.2)	5.0 (3.7)	-3.4 (3.5)	▲75.4 (8.5)	▲78.6 (10.4)	▲86.1 (9.5)	▲70.6 (11.7)
**T5**	**A1**	-12.1 (18.0)	-3.2 (8.6)	11.1 (17.0)	▼12.5 (2.8)	14.9 (10.3)	▲25.7 (17.7)	▲37.4 (16.0)	▲27.3 (18.8)	34.2 (20.7)	5.5 (7.0)	▼7.5 (1.8)	-2.2 (7.0)	▲72.1 (11.6)	▲68.6 (13.7)	▲69.0 (15.3)	▲58.1 (11.3)
**T6**	**A1**	-2.4 (16.0)	-0.6 (11.2)	6.4 (13.0)	15.2 (2.7)	▼9.0 (12.7)	29.0 (10.1)	▼33.9 (14.6)	30.7 (13.0)	39.7 (12.5)	5.3 (5.4)	▲9.1 (3.1)	-4.2 (3.2)	▼30.5 (17.3)	54.3 (12.0)	63.6 (9.9)	61.1 (11.6)
	**A2**	11.8 (14.3)	-2.4 (8.5)	6.5 (15.2)	14.8 (2.7)	▼8.4 (11.4)	30.9 (10.5)	▼31.6 (16.7)	30.2 (14.2)	42.0 (13.6)	3.5 (5.0)	▲9.8 (4.3)	-4.5 (4.5)	▼28.9 (16.7)	56.6 (14.2)	63.9 (10.7)	59.8 (9.8)
	**A3**	-7.0 (21.6)	0.5 (10.6)	10.9 (14.1)	▲16.1 (2.7)	▼9.7 (12.7)	27.0 (7.9)	▼29.8 (15.3)	▼21.7 (14.9)	28.6 (16.7)	4.6 (5.0)	▲8.6 (2.1)	-1.2 (5.4)	▼34.8 (14.8)	51.5 (9.5)	58.8 (11.4)	54.7 (12.4)
**T7**	**A1**	-5.3 (9.1)	▼-0.6 (10.2)	▼10.1 (13.3)	15.3 (1.6)	7.0 (11.5)	▼29.0 (7.9)	35.5 (11.9)	33.5 (13.2)	43.0 (16.2)	▲5.9 (4.0)	7.7 (3.0)	-4.4 (4.7)	28.6 (18.0)	53.3 (12.8)	62.0 (10.0)	▼56.2 (11.1)
	**A2**	1.1 (14.7)	▼-1.0 (9.6)	▼12.0 (10.7)	14.9 (2.1)	7.2 (10.9)	▼28.9 (6.0)	34.8 (12.4)	32.9 (13.2)	41.1 (14.9)	4.4 (4.0)	7.5 (2.8)	-3.9 (4.6)	31.4 (11.6)	52.3 (11.0)	60.3 (10.2)	▼53.8 (11.2)
	**A3**	-4.4 (15.2)	▼-0.7 (11.8)	▼12.3 (9.4)	▲16.3 (2.1)	5.0 (9.8)	▼27.3 (8.5)	▼31.9 (13.2)	26.7 (14.1)	37.3 (17.5)	▲7.2 (4.5)	8.2 (2.9)	-3.7 (4.7)	28.2 (15.4)	47.0 (10.4)	▼57.6 (8.1)	▼52.3 (8.3)
**T8**	**A1**	▲9.7 (14.6)	▼-6.1 (7.2)	1.2 (12.7)	▲16.2 (1.8)	▼4.0 (8.1)	▲28.5 (8.5)	39.9 (11.8)	35.6 (11.5)	46.4 (11.0)	5.7 (5.4)	▲7.4 (3.8)	-4.7 (2.8)	54.5 (13.6)	▲61.9 (7.0)	64.9 (7.4)	56.2 (7.3)
**T9**	**A1**	3.4 (18.3)	-8.0 (6.4)	▲6.3 (14.1)	17.0 (3.6)	10.6 (7.3)	23.7 (16.3)	▼37.9 (15.7)	▼28.6 (11.8)	▼33.2 (11.6)	▲6.6 (4.3)	▲8.2 (2.8)	▲-0.6 (3.4)	59.6 (8.8)	53.8 (7.6)	▼51.0 (8.0)	▼35.4 (9.3)
**T10**	**A1**	-10.1 (12.4)	▼-1.7 (5.5)	13.0 (13.7)	14.8 (1.5)	12.3 (7.3)	▼22.0 (9.9)	32.7 (11.9)	25.7 (10.3)	31.2 (9.7)	▲6.6 (6.0)	▲9.1 (2.6)	-0.3 (3.7)	41.1 (12.3)	50.9 (8.3)	▼53.2 (10.0)	▼41.9 (9.2)
**T11**	**A1**	-13.6 (14.3)	2.0 (7.0)	13.2 (9.6)	14.0 (2.0)	12.0 (11.5)	28.9 (7.1)	38.4 (8.4)	28.3 (7.7)	31.9 (9.5)	▲3.9 (2.9)	▲7.0 (2.5)	-1.3 (3.2)	▼43.5 (11.2)	58.6 (11.6)	66.2 (14.7)	54.9 (16.6)
	**A2**	-15.8 (16.2)	2.5 (6.4)	10.9 (12.6)	13.6 (2.2)	11.5 (8.9)	30.6 (7.4)	36.4 (8.3)	27.2 (9.0)	29.2 (12.4)	2.0 (3.6)	6.3 (2.7)	-1.3 (4.1)	55 (7.6)	61.8 (13.5)	70.5 (14.6)	58.3 (18.4)

Table S2: Mean (SD) of the mean angles (deg) for each joint and task performed with ADs. Statistically significant differences with standard products are indicated: ▲ for higher values when using ADs, ▼ for lower values when using ADs. Joints, tasks and products labelled as in Figure 3. Positive values for flexion, abduction of fingers and palmar deviation of thumb.

	IP1_F	MCP1_F	CMC1_F	CMC1_A	PalmAr	MCP2_F	MCP3_F	MCP4_F	MCP5_F	MCP2-3_A	MCP3-4_A	MCP4-5_A	PIP2_F	PIP3_F	PIP4_F	PIP5_F
**T1**	50.9 (24.9)	18.8 (6.6)	34.2 (14.7)	14.7 (5.5)	26.1 (5.6)	38.9 (7.6)	42.4 (7.3)	34.0 (9.8)	38.5 (13.0)	13.5 (5.1)	9.4 (4.3)	11.0 (5.0)	69.9 (13.6)	61.8 (18.4)	60.9 (22.0)	51.5 (28.6)
**T2**	68.6 (21.3)	24.9 (8.1)	63.2 (24.3)	14.1 (2.8)	30.5 (9.5)	42.7 (11.9)	54.8 (11.4)	48.7 (7.9)	55.1 (10.4)	15.8 (3.8)	11.1 (2.7)	13.6 (4.9)	67.6 (10.2)	71.9 (13.1)	81.4 (17.2)	77.0 (23.9)
**T3**	35.4 (13.1)	14.7 (12.3)	23.1 (9.0)	15.3 (2.9)	13.8 (5.4)	30.3 (6.8)	26.1 (8.2)	28.1 (12.8)	26.9 (15.4)	24.4 (8.2)	13.0 (7.1)	8.5 (3.1)	25.2 (5.6)	28.6 (9.4)	28.4 (7.7)	24.7 (13.6)
**T4**	32.9 (16.1)	15.3 (5.2)	32.7 (8.9)	13.3 (3.5)	22.0 (5.8)	32.4 (9.2)	40.5 (16.9)	44.3 (15.7)	54.7 (15.5)	17.9 (8.5)	9.0 (3.0)	14.3 (5.3)	49.0 (10.0)	47.2 (11.5)	46.4 (14.3)	35.2 (19.7)
**T5**	19.8 (7.8)	10.6 (4.4)	27.4 (9.4)	14.5 (3.4)	20.9 (6.6)	20.6 (6.0)	27.4 (9.0)	20.9 (8.6)	39.4 (12.6)	10.5 (5.3)	8.9 (3.3)	12.2 (5.9)	38.5 (10.5)	33.0 (11.9)	36.6 (16.4)	35.6 (18.7)
**T6**	40.3 (19.3)	14.7 (6.3)	30.3 (10.1)	11.7 (4.4)	26.2 (10.0)	28.3 (7.2)	46.7 (12.7)	46.5 (13.9)	55.2 (14.9)	13.0 (5.8)	8.2 (1.7)	12.6 (2.8)	49.8 (12.6)	56.1 (12.5)	67.6 (14.4)	61.8 (21.2)
**T7**	37.4 (23.7)	18.4 (6.4)	30.7 (9.3)	10.7 (3.2)	23.0 (5.2)	31.6 (9.5)	38.7 (13.0)	39.0 (12.9)	52.7 (12.6)	10.9 (2.6)	7.9 (2.9)	14.2 (3.0)	48.3 (20.1)	52.3 (11.1)	73.2 (11.4)	74.4 (16.3)
**T8**	20.3 (13.5)	12.5 (4.7)	23.2 (9.8)	4.1 (2.0)	14.4 (5.7)	19.9 (8.7)	29.9 (12.6)	38.9 (12.4)	49.4 (13.9)	8.3 (4.8)	6.8 (2.7)	10.4 (3.2)	30.7 (14.2)	33.0 (17.0)	40.6 (17.2)	41.1 (18.9)
**T9**	37.6 (20.5)	21.2 (10.9)	31.2 (8.3)	7.6 (2.9)	16.8 (5.1)	32.6 (7.9)	47.1 (9.2)	48.2 (8.4)	53.9 (11.6)	12.1 (5.8)	11.9 (3.5)	10.5 (4.2)	45.0 (6.7)	42.5 (14.6)	45.9 (14.2)	40.5 (15.5)
**T10**	42.8 (16.1)	18.0 (5.1)	32.5 (13.1)	10.2 (3.2)	24.9 (6.8)	41.1 (7.9)	43.6 (7.1)	49.7 (11.3)	65.3 (14.5)	17.6 (4.5)	9.5 (2.2)	17.7 (5.8)	42.7 (14.1)	48.8 (8.6)	64.6 (10.9)	60.0 (13.4)
**T11**	70.0 (25.0)	21.5 (6.7)	32.7 (10.9)	9.7 (3.4)	22.4 (8.5)	30.6 (4.3)	38.8 (8.2)	39.8 (8.8)	42.0 (13.8)	15.6 (5.9)	10.1 (2.3)	10.0 (3.6)	46.4 (9.1)	47.5 (16.0)	55.1 (19.7)	46.1 (18.2)

Table S3: Mean (SD) of the ROM (deg) for each joint and task performed with normal products. Joints, tasks and products labelled as in Figure 3. Positive values for flexion, abduction of fingers and palmar deviation of thumb.

		IP1_F	MCP1_F	CMC1_F	CMC1_A	PalmAr	MCP2_F	MCP3_F	MCP4_F	MCP5_F	MCP2-3_A	MCP3-4_A	MCP4-5_A	PIP2_F	PIP3_F	PIP4_F	PIP5_F
**T1**	**A1**	50.6 (21.5)	22.9 (11.0)	31.4 (16.3)	13.2 (3.2)	25.3 (8.4)	33.3 (8.0)	37.6 (13.5)	40.5 (14.6)	52.4 (15.6)	13.4 (4.2)	10 (2.7)	15.8 (5.8)	▼46.3 (17.8)	55.1 (13.4)	67.7 (14.1)	65.1 (13.9)
**T2**	**A1**	50.7 (24.3)	29.2 (11.5)	▼40.1 (14.3)	12 (2.5)	27.7 (7.0)	37.1 (15.9)	45.4 (16.7)	45.9 (8.6)	52.8 (6.4)	20.9 (9.5)	11.6 (3.1)	12.8 (2.4)	58.6 (20.2)	▼58.5 (7.8)	▼63.3 (10.0)	▼54.3 (14.4)
	**A2**	56.9 (18.2)	24.6 (8.0)	52.7 (24.1)	14.3 (3.7)	28.3 (5.9)	33.3 (10.8)	50.8 (13.3)	52.0 (11.3)	60.3 (14.7)	18 (3.9)	12.4 (3.5)	14.7 (4.1)	59.1 (17.8)	67.1 (14.9)	76.1 (18.3)	78.8 (24.9)
**T3**	**A1**	19.7 (11.3)	15.8 (8.7)	25.1 (7.4)	▼8.7 (2.7)	12.8 (3.0)	38.6 (15)	▲52.1 (14.8)	▲54.2 (9.0)	▲64.4 (7.0)	▼11.4 (6.8)	▼6.5 (2.4)	12.4 (3.8)	▲72.8 (15.8)	▲77.7 (9.8)	▲82.4 (13.2)	▲64.6 (17.4)
**T4**	**A1**	35.3 (13.5)	20.1 (10.9)	26.2 (14.0)	▼9.2 (3.7)	16.3 (6.5)	42.4 (11.6)	52.5 (12.8)	51.7 (10.7)	54.5 (11.6)	▼8.2 (2.1)	7.3 (2.8)	11.4 (3.2)	▲68.0 (8.5)	▲75.8 (13.7)	▲87.9 (16.0)	▲73.1 (19.9)
**T5**	**A1**	33.1 (18.5)	14.9 (10.5)	24.9 (11.6)	▼8.1 (2.2)	17.5 (7.7)	30.2 (14.5)	▲38.4 (15.1)	▲38.5 (16.0)	45.1 (18.2)	9.8 (4.3)	6.0 (1.9)	11.2 (5.3)	▲68.3 (9.7)	▲68.9 (11.5)	▲73.2 (16.8)	▲64.4 (19.4)
**T6**	**A1**	▼22.5 (12.0)	15.4 (8.6)	25.5 (9.2)	10.4 (4.0)	20.6 (7.4)	27.2 (5.7)	▼34.6 (9.8)	39.1 (9.5)	47.7 (12.1)	12.7 (6.1)	7.6 (2.0)	11.3 (3.2)	▼29.5 (15.0)	50.4 (13.2)	64.6 (14.1)	62.3 (15.5)
	**A2**	30.2 (12.2)	12.9 (4.9)	22.2 (5.9)	10.9 (3.9)	18.1 (4.2)	28.9 (5.2)	▼34.3 (9.2)	40.1 (10.0)	48.9 (11.7)	12.2 (3.7)	9.4 (3.5)	10.9 (3.3)	▼32.2 (17.2)	48.1 (19.9)	60.0 (20.7)	59.6 (22.4)
	**A3**	31.8 (13.2)	16.1 (7.6)	25.5 (8.9)	11.2 (4.2)	17.0 (5.7)	23.0 (4.6)	▼31.1 (8.1)	▼30.9 (10.2)	43.0 (12.3)	13.1 (6.1)	6.6 (2.2)	11.6 (5.5)	▼33.7 (9.6)	45.1 (15.5)	54.9 (20.8)	54.5 (19.2)
**T7**	**A1**	27.3 (14.7)	14.2 (5.1)	29.0 (14.6)	11.3 (2.7)	22.1 (5.8)	26.1 (11.5)	37.5 (11.3)	45.3 (13.2)	52.5 (11.1)	13.9 (4.6)	8.8 (2.4)	13.1 (2.8)	40.5 (13.9)	51.7 (15.0)	64.7 (14.8)	▼60.3 (19.5)
	**A2**	26.5 (17.5)	15.2 (5.4)	25.6 (10.5)	11.9 (4.5)	19.7 (5.5)	25.5 (7.5)	36.4 (11.3)	45.1 (14.0)	52.2 (15.1)	13.8 (4.4)	8.7 (2.8)	12.0 (2.6)	40.1 (15.2)	52.1 (13.3)	66.4 (12.6)	▼62.0 (15.5)
	**A3**	26.7 (9.9)	16.9 (6.1)	23.8 (8.4)	13.0 (2.9)	20.3 (6.5)	28.8 (9.5)	31.3 (10.1)	33.7 (10.9)	46.3 (12.7)	▲18.0 (6.1)	7.6 (1.4)	▼11.0 (2.6)	41.0 (13.2)	51.5 (10.1)	▼63.6 (8.7)	60.8 (10.0)
**T8**	**A1**	▲35.1 (22.5)	17.0 (7.8)	21.5 (13.6)	8.1 (3.8)	▲24.3 (7.1)	▲33.3 (10.4)	▲47.7 (13.2)	▲48.3 (7.3)	52.4 (8.0)	12.3 (4.9)	9.0 (3.1)	9.9 (1.7)	42.1 (8.4)	38.2 (16.1)	38.4 (15.5)	33.8 (13.0)
**T9**	**A1**	32.5 (16.0)	16.4 (10.2)	25.6 (11.0)	9.7 (2.7)	16.3 (5.4)	37.2 (8.3)	43.4 (16.7)	40.7 (16.9)	42.9 (16.2)	8.9 (4.3)	▼6.2 (3.1)	7.3 (2.2)	43.1 (10.0)	36.3 (10.8)	▼33.9 (8.9)	▼21.8 (5.5)
**T10**	**A1**	38.8 (10.6)	19.8 (7.4)	32.5 (11.5)	10.8 (3.6)	24.7 (7.4)	39.8 (10.1)	44.3 (11.4)	50.2 (12.6)	60.6 (10.6)	18.4 (4.7)	9.4 (2.8)	▼14.2 (5.4)	47.2 (8.9)	▼39.7 (9.5)	▼42.6 (7.6)	▼38.9 (10.5)
**T11**	**A1**	60.3 (21.3)	24.4 (7.0)	32.8 (11.6)	9.8 (2.9)	▲30.8 (8.0)	33.6 (7.0)	49.3 (18.6)	45.9 (13.3)	48.8 (10.5)	17.8 (8.5)	9.8 (3.9)	10.2 (2.9)	51.2 (14.6)	50.0 (16.6)	54.5 (18.2)	53.8 (15.8)
	**A2**	53.7 (18.3)	18.5 (5.8)	30.4 (9.1)	10.2 (2.5)	24.5 (7.9)	30.5 (7.0)	44.2 (9.4)	44.1 (7.8)	44.3 (10.4)	16.2 (4.2)	10.1 (2.4)	11.6 (3.7)	54.3 (12.4)	55.4 (11.8)	61.3 (14.6)	50.3 (13.6)

Table S4: Mean (SD) of the ROM (deg) for each joint and task performed with ADs. Statistically significant differences with standard products are indicated: ▲ for higher values when using ADs, ▼ for lower values when using ADs. Joints, tasks and products labelled as in Figure 3. Positive values for flexion, abduction of fingers and palmar deviation of thumb.

	IP1_F	MCP1_F	CMC1_F	CMC1_A	PalmAr	MCP2_F	MCP3_F	MCP4_F	MCP5_F	MCP2-3_A	MCP3-4_A	MCP4-5_A	PIP2_F	PIP3_F	PIP4_F	PIP5_F
**T1**	8.7 (6.3)	3.8 (2.7)	12.5 (5.0)	2.6 (1.9)	9.4 (3.9)	11.1 (7.0)	14.4 (6.4)	9.9 (3.9)	8.2 (6.3)	6.5 (2.4)	4.3 (1.2)	4.7 (1.8)	21.9 (5.1)	15.6 (6.7)	11.5 (8.1)	9.5 (7.5)
**T2**	28.8 (10.5)	17.1 (4.6)	31.7 (6.7)	8.3 (1.6)	15.8 (5.9)	23.3 (5.2)	34.5 (7.1)	27.8 (4.9)	28.5 (9.0)	10.0 (1.6)	7.6 (1.7)	8.5 (2.3)	24.9 (7.9)	28.1 (7.8)	31.9 (10.2)	36.7 (12.4)
**T3**	0.4 (0.5)	0.4 (1.2)	2.4 (3.5)	0.1 (0.1)	2.2 (1.9)	0.5 (1.0)	0.6 (0.5)	0.9 (1.4)	1.2 (3.1)	1.0 (0.9)	1.3 (1.1)	1.0 (0.8)	0.1 (0.2)	0.4 (0.5)	0.2 (0.4)	0.3 (0.4)
**T4**	1.0 (2.6)	0.1 (0.2)	1.5 (1.4)	0.1 (0.1)	2.3 (2.3)	0.6 (0.6)	0.6 (1.1)	1.7 (4.2)	1.2 (1.9)	1.0 (0.7)	1.2 (1.5)	1.6 (2.1)	0.2 (0.3)	0.5 (0.7)	0.4 (0.4)	0.2 (0.3)
**T5**	0.4 (0.6)	0.1 (0.1)	1.9 (1.8)	0.1 (0.1)	3.1 (1.8)	0.5 (0.4)	0.8 (0.7)	0.9 (0.8)	1.3 (1.6)	0.7 (0.6)	1.0 (0.5)	2.1 (1.4)	0.2 (0.3)	0.3 (0.5)	0.4 (0.4)	1.0 (0.9)
**T6**	13.2 (9.2)	2.1 (1.6)	11.0 (5.4)	1.9 (2.3)	7.1 (5.2)	5.0 (5.4)	6.2 (5.2)	9.9 (6.3)	8.9 (6.5)	4.2 (2.9)	3.8 (1.5)	3.4 (1.1)	8.5 (10.8)	5.4 (6.1)	4.4 (4.9)	5.2 (4.5)
**T7**	8.3 (7.1)	2.7 (2.0)	11.1 (6.4)	1.4 (1.6)	8.5 (3.4)	5.6 (5.7)	3.5 (3.1)	6.1 (4.8)	7.3 (5.2)	4.5 (1.9)	3.6 (2.1)	3.4 (1.7)	5.0 (4.7)	4.3 (5.9)	3.6 (3.5)	7.1 (6.8)
**T8**	1.7 (3.1)	0.4 (0.3)	4.0 (3.4)	0.2 (0.2)	3.4 (2.0)	1.1 (1.2)	1.5 (1.8)	3.6 (4.5)	3.9 (3.7)	0.7 (0.4)	1.6 (0.9)	1.8 (1.3)	1.2 (1.4)	1.5 (2.4)	1.6 (1.9)	1.9 (2.1)
**T9**	1.3 (1.5)	1.7 (2.0)	6.5 (3.6)	0.6 (0.7)	3.4 (1.9)	2.0 (1.6)	3.4 (4.6)	2.7 (4.2)	2.5 (4.5)	1.2 (1.5)	1.7 (1.4)	1.1 (0.8)	1.2 (1.5)	1.6 (2.3)	1.2 (1.3)	2.1 (3.5)
**T10**	8.0 (5.2)	3.1 (3.8)	13.6 (5.2)	2.5 (2.3)	8.9 (5.5)	9.8 (5.2)	10.5 (6.3)	10.3 (4.9)	13.0 (7.7)	6.3 (3.1)	3.8 (0.9)	5.1 (1.7)	9.6 (6.4)	5.3 (6.7)	6.0 (6.9)	6.1 (6.7)
**T11**	24.0 (14.0)	9.8 (4.8)	15.6 (5.0)	2.5 (1.4)	9.2 (4.8)	11.6 (7.0)	12.1 (6.8)	13.4 (5.6)	12.1 (5.8)	6.3 (2.2)	5.2 (2.0)	4.4 (1.3)	17.1 (8.0)	10.1 (6.3)	9.8 (5.5)	8.7 (6.7)

Table S5: Mean (SD) of the median velocity (deg/s) for each joint and task performed with normal products. Joints, tasks and products labelled as in Figure 3. Positive values for flexion, abduction of fingers and palmar deviation of thumb.

.

Task	Product	IP1_F	MCP1_F	CMC1_F	CMC1_A	PalmAr	MCP2_F	MCP3_F	MCP4_F	MCP5_F	MCP2-3_A	MCP3-4_A	MCP4-5_A	PIP2_F	PIP3_F	PIP4_F	PIP5_F
**T1**	**A1**	6.8 (6.6)	3.7 (4.4)	11.5 (6.8)	1.9 (2.1)	10.0 (2.7)	▼4.5 (4.1)	▼5.2 (4.8)	6.2 (5.6)	8.7 (6.7)	4.1 (2.2)	3.7 (2.2)	4.9 (2.4)	▼4.8 (6.8)	▼3.0 (4.0)	4.6 (6.0)	8.1 (8.8)
**T2**	**A1**	22.0 (13.0)	▼12.6 (6.3)	25.1 (7.7)	▼4.0 (2.5)	16.3 (4.7)	16.4 (7.4)	▼22.3 (11.7)	24.8 (11.1)	26.9 (10.0)	10.0 (4.3)	7.4 (1.9)	8.2 (2.3)	14.1 (11.2)	20.5 (13.2)	24.3 (10.5)	26.3 (9.6)
	**A2**	25.4 (7.3)	16.2 (4.9)	29.4 (9.5)	6.7 (2.5)	13.1 (5.6)	20.1 (6.6)	26.6 (7.5)	23.0 (6.6)	23.6 (8.3)	12.4 (3.4)	8.5 (2.7)	7.8 (1.4)	21.2 (7.7)	23.2 (8.5)	26.7 (8.6)	29.9 (10.1)
**T3**	**A1**	0.0 (0.0)	0.1 (0.1)	1.8 (1.6)	0.2 (0.2)	1.2 (2.0)	0.0 (0.0)	▼0.1 (0.2)	0.1 (0.1)	0.0 (0.1)	0.3 (0.3)	▼0.2 (0.2)	▼0.2 (0.2)	0.0 (0.1)	0.0 (0.0)	0.0 (0.0)	0.1 (0.2)
**T4**	**A1**	0.3 (0.8)	0.3 (0.8)	1.1 (2.9)	0.1 (0.3)	0.6 (1.0)	▼0.2 (0.4)	0.3 (0.6)	0.2 (0.4)	0.2 (0.2)	▼0.3 (0.5)	0.4 (0.7)	0.3 (0.4)	0.1 (0.3)	0.0 (0.1)	0.0 (0.1)	0.1 (0.2)
**T5**	**A1**	0.5 (0.8)	0.3 (0.5)	2.6 (2.3)	0.2 (0.2)	2.3 (2.5)	0.3 (0.3)	0.3 (0.4)	0.7 (0.8)	1.2 (1.5)	0.6 (0.7)	0.6 (0.6)	1.3 (1.3)	0.2 (0.4)	0.2 (0.4)	0.3 (0.5)	0.6 (0.8)
**T6**	**A1**	▼2.3 (3.4)	0.6 (0.7)	6.0 (3.5)	0.6 (0.7)	5.6 (2.5)	2.2 (2.4)	5.0 (4.7)	5.1 (6.0)	5.8 (6.7)	3.6 (1.9)	2.9 (1.1)	2.5 (1.3)	1.1 (2.4)	0.9 (1.5)	0.4 (0.3)	1.2 (1.4)
	**A2**	▼2.9 (2.7)	▼0.5 (0.4)	▼5.1 (2.3)	0.4 (0.6)	4.9 (2.2)	2.1 (2.1)	4.1 (4.7)	5.5 (4.7)	3.7 (4.8)	3.8 (1.6)	3.1 (0.9)	2.8 (1.2)	1.1 (2.0)	0.3 (0.3)	0.5 (0.4)	▼0.6 (0.6)
	**A3**	▼3.5 (4.6)	2.0 (3.5)	▼5.0 (2.7)	0.4 (0.6)	5.7 (2.5)	1.8 (2.1)	4.6 (7.0)	4.8 (7.0)	5.2 (6.0)	3.0 (1.8)	2.3 (1.8)	2.4 (1.3)	1.1 (0.8)	0.6 (0.8)	0.6 (0.8)	1.9 (1.9)
**T7**	**A1**	3.7 (3.9)	▼1.1 (1.1)	9.8 (3.7)	0.7 (0.5)	8.3 (2.1)	2.9 (2.3)	4.1 (3.6)	6.3 (5.0)	4.5 (5.2)	4.5 (2.6)	4.0 (2.1)	3.0 (1.6)	3.0 (3.9)	1.4 (2.0)	1.1 (1.5)	3.2 (2.9)
	**A2**	3.4 (3.5)	1.1 (0.9)	7.9 (4.7)	0.8 (1.1)	7.6 (2.5)	4.2 (3.4)	3.6 (3.4)	6.0 (5.2)	4.8 (4.0)	4.3 (2.5)	3.5 (2.2)	2.6 (1.1)	3.9 (5.1)	1.9 (2.5)	2.5 (4.0)	3.4 (3.7)
	**A3**	4.4 (5.5)	2.2 (2.5)	10.5 (3.6)	1.3 (1.2)	7.8 (1.9)	3.4 (2.8)	2.8 (3.2)	▼2.2 (1.4)	▼2.4 (1.2)	4.6 (2.1)	3.1 (1.6)	3.0 (1.3)	3.4 (4.0)	3.2 (4.8)	3.6 (5.1)	4.7 (4.8)
**T8**	**A1**	1.7 (1.4)	0.8 (0.8)	4.0 (2.3)	0.2 (0.1)	3.3 (2.2)	1.6 (1.8)	1.7 (2.2)	1.7 (2.1)	1.4 (1.6)	1.6 (1.4)	1.4 (1.1)	0.9 (0.5)	0.9 (0.8)	0.3 (0.4)	0.1 (0.1)	0.4 (0.5)
**T9**	**A1**	1.6 (2.4)	0.5 (0.5)	5.2 (4.7)	0.4 (0.3)	2.2 (2.0)	2.2 (3.0)	2.9 (3.8)	2.3 (2.7)	2.8 (3.8)	0.9 (0.8)	1.3 (1.2)	1.6 (1.4)	0.7 (1.3)	0.3 (0.4)	0.5 (0.9)	1.1 (1.7)
**T10**	**A1**	9.2 (8.6)	3.9 (4.0)	12.7 (6.8)	2.2 (1.9)	8.8 (4.2)	8.4 (7.3)	10.4 (8.4)	9.0 (7.7)	8.6 (7.2)	5.9 (4.5)	3.6 (1.9)	3.6 (1.9)	6.6 (8.2)	4.1 (7.3)	3.8 (6.4)	5.7 (5.9)
**T11**	**A1**	▼16.2 (8.7)	8.6 (4.2)	14.5 (4.2)	3.0 (1.7)	9.3 (3.6)	9.0 (4.4)	11.7 (5.1)	11.7 (5.7)	10.3 (5.6)	6.4 (2.2)	4.8 (1.9)	3.9 (1.3)	▼10.2 (4.8)	7.7 (5.3)	6.6 (4.4)	7.5 (5.3)
	**A2**	19.6 (10.9)	8.2 (3.4)	16.8 (4.7)	3.3 (1.4)	8.3 (3.6)	10.2 (4.4)	13.5 (6.8)	14.0 (5.2)	11.3 (4.2)	7.3 (2.1)	4.9 (1.8)	4.0 (1.3)	16.7 (7.7)	9.6 (7.3)	7.5 (5.8)	8.7 (4.6)

Table S6: Mean (SD) of the median velocity (deg/s) for each joint and task performed with the ADs. Statistically significant differences with standard products are indicated: ▲ for higher values when using ADs, ▼ for lower values when using ADs. Joints, tasks and products labelled as in Figure 3. Positive values for flexion, abduction of fingers and palmar deviation of thumb.

	IP1_F	MCP1_F	CMC1_F	CMC1_A	PalmAr	MCP2_F	MCP3_F	MCP4_F	MCP5_F	MCP2-3_A	MCP3-4_A	MCP4-5_A	PIP2_F	PIP3_F	PIP4_F	PIP5_F
**T1**	100.1 (38.4)	38.7 (12.0)	58.8 (20.4)	21.1 (7.1)	47.0 (9.6)	72.8 (21.1)	71.8 (19.0)	58.0 (18.0)	53.4 (19.6)	37.1 (9.7)	20.1 (6.5)	21.1 (9.5)	126.1 (29.3)	92.2 (31.4)	88.2 (29.5)	65.8 (24.9)
**T2**	181.8 (59.8)	99.9 (30.7)	170.9 (61.1)	37.1 (8.6)	89.2 (21.3)	131.8 (37.5)	184.5 (38.4)	186.1 (45.8)	202.3 (44.3)	72.8 (26.8)	40.1 (11.5)	41.8 (13.4)	139.2 (30.1)	140.3 (43.9)	157.7 (57.2)	162.9 (74.0)
**T3**	61.0 (29.1)	28.0 (20.8)	40.5 (17.2)	19.8 (4.7)	29.6 (10.1)	64.1 (17.5)	60.7 (13.4)	45.1 (12.0)	36.7 (12.9)	29.6 (11.2)	22.1 (15.4)	18.6 (10.9)	42.7 (10.8)	50.1 (12.1)	52.1 (18.3)	56.8 (34.1)
**T4**	48.0 (19.9)	30.3 (8.6)	48.1 (16.3)	16.7 (3.7)	35.2 (11.6)	61.3 (23.2)	74.1 (39.6)	56.1 (20.2)	61.7 (20.3)	26.8 (7.8)	18.9 (4.9)	22.2 (7.9)	77.2 (33.0)	68.0 (23.9)	66.6 (19.8)	56.0 (24.4)
**T5**	35.0 (14.5)	22.0 (5.9)	47.2 (17.1)	18.5 (5.3)	37.6 (7.3)	43.9 (9.7)	56.9 (14.6)	39.7 (10.3)	58.8 (22.9)	18.1 (5.3)	17.9 (4.9)	22.9 (8.1)	53.5 (9.9)	46.1 (16.4)	57.9 (30.6)	63.9 (25.1)
**T6**	106.8 (47.3)	39.4 (14.2)	68.9 (21.7)	26.0 (8.9)	53.8 (15.0)	63.6 (28.2)	82.7 (27.3)	78.1 (27.7)	89.5 (30.4)	36.1 (16.9)	21.4 (5.0)	21.5 (4.2)	96.1 (29.3)	97.9 (33.7)	113.5 (40.7)	104.6 (38.0)
**T7**	83.5 (45.3)	38.8 (12.4)	71.8 (21.1)	19.6 (8.4)	65.2 (18.0)	77.3 (22.7)	81.0 (26.6)	84.7 (27.1)	103.0 (31.7)	40.0 (8.6)	25.5 (9.3)	29.4 (9.9)	125.6 (39.0)	96.2 (29.8)	134.3 (36.1)	146.3 (56.3)
**T8**	42.1 (28.7)	29.7 (9.6)	48.9 (19.1)	10.8 (3.0)	33.7 (10.7)	48.9 (15.4)	74.5 (23.1)	68.9 (22.4)	76.5 (25.7)	16.7 (4.8)	16.5 (4.9)	18.6 (6.1)	57.4 (28.7)	60.2 (23.6)	69.0 (27.7)	72.4 (34.9)
**T9**	92.7 (64.9)	36.4 (14.2)	63.5 (21.6)	15.4 (4.9)	35.5 (6.5)	69.0 (20.8)	85.8 (32.3)	86.9 (30.2)	88.2 (26.3)	23.7 (10.7)	22.7 (6.4)	17.8 (3.6)	88.8 (24.5)	60.5 (18.3)	69.1 (19.8)	69.8 (24.9)
**T10**	120.4 (40.1)	48.9 (15.5)	91.5 (38.3)	24.9 (8.4)	67.0 (24.1)	98.1 (22.0)	123.9 (30.0)	129.9 (48.6)	143.9 (47.9)	56.7 (25.5)	28.5 (10.3)	38.5 (13.0)	124.0 (25.2)	116.0 (34.5)	136.1 (34.3)	152.0 (37.4)
**T11**	156.1 (54.9)	62.6 (27.7)	80.1 (20.2)	26.8 (6.5)	51.5 (28.5)	81.5 (23.8)	99.0 (24.2)	95.8 (25.0)	81.6 (29.9)	46.9 (13.0)	25.8 (5.8)	21.1 (6.1)	135.6 (26.8)	112.5 (40.2)	119.5 (39.0)	81.5 (36.3)

Table S7: Mean (SD) of the P95 velocity (deg/s) for each joint and task when performed with normal products. Joints, tasks and products labelled as in Figure 3. Positive values for flexion, abduction of fingers and palmar deviation of thumb.

		IP1_F	MCP1_F	CMC1_F	CMC1_A	PalmAr	MCP2_F	MCP3_F	MCP4_F	MCP5_F	MCP2-3_A	MCP3-4_A	MCP4-5_A	PIP2_F	PIP3_F	PIP4_F	PIP5_F
**T1**	**A1**	111.3 (39.6)	44.0 (21.8)	73.8 (37.4)	22.5 (7.5)	57.3 (14. 3)	63.1 (16.6)	74.2 (18.0)	74.2 (20.3)	86.0 (34.6)	31.5 (10.1)	22.7 (7.8)	28.9 (8.6)	82.4 (35.9)	81.0 (13.8)	95.6 (22.0)	▲112.2 (49.1)
**T2**	**A1**	162.8 (78.5)	▼74.5 (35.0)	▼122.2 (51.3)	▼25.7 (6.8)	74.6 (19.2)	▼94.6 (36.3)	▼124.3 (45.5)	▼129.9 (50.1)	▼143.9 (50.0)	▼52.4 (19.2)	37.7 (14.0)	39.9 (11.3)	119.0 (40.6)	146.7 (54.8)	157.6 (45.3)	159.6 (44.9)
	**A2**	180.3 (52.0)	89.1 (17.5)	144.4 (54.2)	37.2 (11.1)	83.6 (14.5)	112.4 (30.5)	156.7 (29.9)	157.1 (42.8)	173.1 (37.5)	72.9 (23.4)	41.3 (13.5)	41.3 (8.4)	122.8 (33.5)	142.7 (17.5)	161.7 (23.7)	174.9 (51.7)
**T3**	**A1**	36.7 (12.9)	28.2 (9.8)	53.7 12.9)	▼15.9 (4.1)	32.3 (10.6)	78.5 (28.8)	▲103.2 (40.8)	▲95.7 (32.0)	▲97.6 (31.2)	▼20.7 (6.3)	18.4 (7.4)	21.5 (7.8)	▲115.5 (44.7)	▲110.7 (35.0)	▲119.4 (37.5)	▲98.6 (39.4)
**T4**	**A1**	60.0 (21.0)	41.1 (15.7)	51.6 (23.1)	16.2 (5.3)	30.9 (10.1)	65.0 (18.9)	79.4 (28.3)	67.2 (15.3)	68.5 (20.5)	▼18.2 (4.5)	15.3 (4.0)	18.6 (6.6)	89.1 (28.1)	▲95.2 (21.4)	▲113.1 (28.2)	▲96.5 (33.5)
**T5**	**A1**	51.1 (26.4)	25.9 (9.1)	46.2 (15.8)	16.6 (3.7)	36.2 (18.5)	58.2 (21.8)	64.4 (20.6)	55.1 (22.2)	61.2 (23.4)	16.8 (5.0)	13.8 (3.0)	22.3 (14.8)	▲92.8 (27.0)	▲93.7 (25.4)	101.9 (32.7)	89.1 (32.3)
**T6**	**A1**	▼60.4 (25.7)	39.5 (15.1)	▼51.6 (14.4)	▼17.7 (5.6)	48.2 (17.6)	52.8 (21.9)	▼63.1 (22.2)	62.9 (14.3)	▼69.9 (21.3)	31.8 (16.6)	21.9 (7.4)	21.1 (5.1)	▼62.2 (41.7)	95.2 (27.2)	111.7 (29.3)	110.3 (30.1)
	**A2**	▼59.9 (22.0)	29.4 (9.2)	▼51.5 (13.7)	18.8 (8.4)	47.4 (10.6)	58.6 (26.7)	▼70.3 (26.2)	63.9 (15.5)	▼68.2 (14.7)	33.6 (14.3)	21.8 (7.0)	22.3 (9.1)	▼58.0 (26.1)	94.1 (36.3)	116.2 (45.8)	112.6 (46.6)
	**A3**	▼62.7 (33.5)	38.9 (13.2)	▼50.2 (16.7)	▼16.9 (5.6)	46.9 (11.7)	57.2 (18.5)	70.1 (23.7)	60.6 (20.2)	74.5 (24.1)	32.8 (17.8)	18.9 (7.4)	23.7 (10.7)	▼67.0 (19.0)	90.7 (25.4)	106.5 (31.2)	103.0 (37.2)
**T7**	**A1**	80.5 (33.2)	38.7 (11.3)	66.9 (26.3)	20.0 (5.3)	62.8 (14.7)	▼60.6 (21.1)	75.1 (21.8)	77.4 (28.9)	89.7 (33.7)	34.9 (10.9)	26.0 (6.6)	28.4 (9.9)	120.2 (43.6)	115.1 (35.3)	126.3 (41.3)	▼113.3 (38.9)
	**A2**	72.1 (38.9)	33.8 (10.5)	63.6 (16.2)	19.4 (6.6)	58.2 (14.3)	63.5 (20.5)	76.5 (22.5)	75.9 (26.5)	83.3 (25.0)	39.7 (13.2)	28.2 (8.4)	26.3 (9.0)	▼94.8 (34.0)	115.9 (35.8)	136.0 (45.7)	127.5 (45.1)
	**A3**	95.7 (36.0)	41.4 (12.2)	68.0 (17.8)	26.0 (5.2)	68.5 (13.6)	83.3 (25.7)	97.0 (16.5)	83.0 (29.9)	91.0 (32.9)	45.9 (13.7)	28.8 (8.8)	27.2 (10.1)	131.6 (41.8)	▲134.9 (39.3)	136.2 (38.8)	124.5 (29.5)
**T8**	**A1**	▲83.4 (30.1)	36.9 (10.9)	54.3 (16.7)	14.4 (4.0)	▲50.9 (11.5)	▲89.8 (23.8)	▲107.5 (27.9)	89.6 (23.5)	79.3 (21.3)	▲34.5 (10.8)	▲23.5 (7.7)	21.3 (5.1)	▲94.8 (19.5)	78.2 (25.2)	83.3 (24.8)	89.1 (32.5)
**T9**	**A1**	64.4 (26.3)	35.5 (15.1)	62.7 (23.5)	17.2 (5.6)	35.5 (11.1)	96.6 (28.3)	121.2 (43.7)	97.9 (40.1)	92.9 (41.1)	21.6 (6.1)	16.0 (8.4)	19.1 (6.7)	101.9 (27.4)	▲88.4 (30.0)	83.9 (26.1)	59.4 (22.8)
**T10**	**A1**	128.2 (60.0)	43.8 (16.0)	77.7 (21.9)	23.0 (8.4)	56.5 (19.6)	92.2 (31.4)	107.5 (28.7)	103.7 (29.7)	120.0 (39.4)	57.4 (27.6)	28.4 (8.1)	35.0 (17.1)	103.6 (32.3)	▼86.3 (29.4)	▼100.0 (33.7)	▼107.0 (33.5)
**T11**	**A1**	146.7 (57.8)	67.2 (29.3)	82.6 (29.8)	24.9 (5.7)	63.9 (11.6)	89.2 (22.0)	98.8 (20.2)	96.1 (21.3)	91.1 (22.4)	57.1 (22.7)	25.9 (5.8)	22.3 (7.1)	116.1 (26.3)	114.5 (30.5)	114.3 (31.1)	104.9 (17.8)
	**A2**	143.5 (53.3)	55.8 (19.8)	83.7 (18.4)	31.1 (9.4)	48.4 (15.4)	78.1 (20.2)	117.3 (25.3)	110.0 (23.5)	90.9 (22.1)	46.7 (14.3)	26.1 (4.7)	23.1 (5.9)	137.2 (34.3)	135.3 (45.0)	130.1 (50.4)	97.4 (45.5)

Table S8: Mean (SD) of the percentile P95 values of velocities (deg/s) for each joint and task performed with the ADs. Statistically significant differences with the standard products are indicated: ▲ for higher values when using ADs, ▼ for lower values when using ADs. Joints, tasks and products labelled as in Figure 3. Positive values for flexion, abduction of fingers and palmar deviation of thumb.
